# Sense of Agency Beyond Sensorimotor Process: Decoding Self-Other Action Attribution in the Human Brain

**DOI:** 10.1093/cercor/bhaa028

**Published:** 2020-03-03

**Authors:** Ryu Ohata, Tomohisa Asai, Hiroshi Kadota, Hiroaki Shigemasu, Kenji Ogawa, Hiroshi Imamizu

**Affiliations:** 1 Department of Psychology, Graduate School of Humanities and Sociology, The University of Tokyo, Bunkyo-ku, Tokyo 113-0033, Japan; 2 Department of Cognitive Neuroscience, Cognitive Mechanisms Laboratories, Advanced Telecommunications Research Institute International (ATR), Keihanna Science City, Kyoto 619-0288, Japan; 3 School of Information, Kochi University of Technology, Kami, Kochi 782-8502, Japan; 4 Research Institute, Kochi University of Technology, Kami, Kochi 782-8502, Japan; 5 Department of Psychology, Graduate School of Humanities and Human Sciences, Hokkaido University, Sapporo, Hokkaido 060-0810, Japan

**Keywords:** functional magnetic resonance imaging, inferior parietal lobe, multivoxel pattern analysis, sense of agency, supramarginal gyrus

## Abstract

The sense of agency is defined as the subjective experience that “I” am the one who is causing the action. Theoretical studies postulate that this subjective experience is developed through multistep processes extending from the sensorimotor to the cognitive level. However, it remains unclear how the brain processes such different levels of information and constitutes the neural substrates for the sense of agency. To answer this question, we combined two strategies: an experimental paradigm, in which self-agency gradually evolves according to sensorimotor experience, and a multivoxel pattern analysis. The combined strategies revealed that the sensorimotor, posterior parietal, anterior insula, and higher visual cortices contained information on self-other attribution during movement. In addition, we investigated whether the found regions showed a preference for self-other attribution or for sensorimotor information. As a result, the right supramarginal gyrus, a portion of the inferior parietal lobe (IPL), was found to be the most sensitive to self-other attribution among the found regions, while the bilateral precentral gyri and left IPL dominantly reflected sensorimotor information. Our results demonstrate that multiple brain regions are involved in the development of the sense of agency and that these show specific preferences for different levels of information.

## Introduction

How the brain makes us aware of our selfhood, as an individual separate from other individuals, is a long-standing question in the field of neuroscience. The sense of agency is defined as a subjective experience that “I” am the one who is causing or generating an action (Gallagher 2000; Haggard 2017). This definition illustrates the interaction between body and environment (i.e., a sensorimotor process), specifying the self as the subject of action and perception (Legrand 2007; Legrand and Ruby 2009; Christoff et al. 2011). Previous neuroimaging studies have reported multiple brain regions associated with the sense of agency, such as the supplementary motor area (Tsakiris et al. 2010; Yomogida et al. 2010; Miele et al. 2011), the cerebellum (Blakemore et al. 2001; Yomogida et al. 2010), the posterior parietal cortex (Farrer and Frith 2002; Farrer et al. 2003, 2008; Ogawa and Inui 2007; Schnell et al. 2007; Spengler et al. 2009; Yomogida et al. 2010; Miele et al. 2011; Nahab et al. 2011; Chambon et al. 2013; Fukushima et al. 2013; Beyer et al. 2018), the lateral prefrontal cortex (Schnell et al. 2007; Nahab et al. 2011; Chambon et al. 2013), the higher visual cortex (Astafiev et al. 2004; David et al. 2007; Yomogida et al. 2010), and the insula (Farrer et al. 2003; Tsakiris et al. 2010; Fukushima et al. 2013) (see also reviews and meta-analysis studies in David et al. 2008; Miele et al. 2011; Sperduti et al. 2011). Most of these studies manipulated the discrepancy between participants’ own actions and the sensory consequences for controlling the participants’ attribution of the observed action to the self or to another. Then, they made contrasts between the self-attribution condition (the observed action is attributed to oneself) and the other-attribution condition (the action is attributed to another). These studies indicated that the reported regions are recruited at a certain stage of the sense of agency; however, it has not been clarified how the brain regions are involved in the process to develop sensorimotor information into agency attribution.

**Figure 1:**
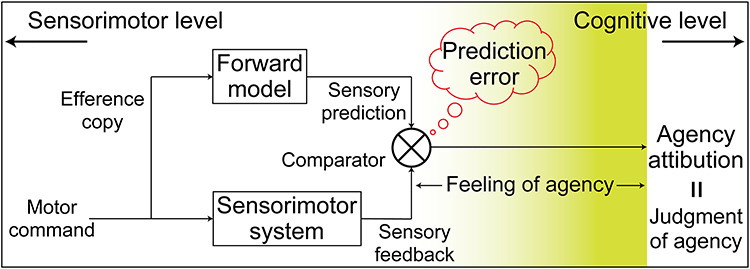
Multistep processes behind the sense of agency extending from lower sensorimotor to higher cognitive level processing. This schematic is an overview combing the two influential theories of the sense of agency: 1) the comparator model (Miall and Wolpert 1996; Blakemore et al. 1998, 2000) and 2) the two-step account of agency (Synofzik et al. 2008, 2013). Considering the two theoretical models together, agency attribution is achieved through the multistep processes extending from lower sensorimotor to higher cognitive level. The hypothesis in the current study is that some brain regions represent the immediate output of sensorimotor processing, while others represent the information directly leading to agency attribution. The background yellow gradation depicts the level of the information represented in the brain from sensorimotor to cognitive level, which is the main target of the current study. In Synofzik et al. (2008), the sense of agency encompasses two levels of representation: a nonconceptual feeling and a conceptual judgment of agency. The term “agency attribution” (or “self-other attribution”) in the current study corresponds to a conceptual judgment of agency. We assume that the process leading to the conceptual judgment of agency (not including the judgment process itself) based on the lower sensorimotor information is a nonconceptual “feeling of agency.” We use the term “sense of agency” to include both a nonconceptual feeling and a conceptual judgment of agency.

Theoretical models provide a clue for elucidating the neural process behind the sense of agency grounded on sensorimotor information. The comparator model, one of the most influential models of the sense of agency, has suggested the importance of sensorimotor processing for agency attribution (Fig. 1 left). This model suggests that the brain compares predicted and actual sensory consequences of an action (Miall and Wolpert 1996; Blakemore et al. 1998, 2000). The result of this comparison, known as a prediction error, determines whether people attribute the observed action to their own or to another agent (Blakemore et al. 2000; Frith et al. 2000). As the comparator model highlights the sensorimotor process (i.e., calculation of sensory prediction error), there exists a gap between the sensorimotor process and agency attribution. Synofzik et al. (2008) postulated the necessity of an intermediate process, named a nonconceptual feeling of agency, to take over the outcome of the comparator model and develop the information needed to achieve a conceptual judgment of agency (Fig. 1 right, see also Synofzik et al. 2013). Summarizing the above, the two theoretical models suggest that there need to be multistep processes for the sense of agency extending from the lower sensorimotor to higher cognitive level (Fig. 1).

The current study reflected the theoretical models’ implications in the hypothesis regarding the neural process. Namely, we hypothesized that there exists gradation in neural information from the lower sensorimotor to higher cognitive level (Fig. 1, yellow gradation): Some brain regions preferentially represent the information closely related to agency attribution, while others represent the immediate output of sensorimotor processing. We tested the hypothesis by combining the following two strategies. First, we used an experimental paradigm in which self- or other agency gradually evolves according to the amount of sensorimotor experience (Nahab et al. 2011; Asai 2016). Participants continuously traced a target path by controlling a joystick under ambiguous conditions of agency. That is, we morphed visual feedback of the movement (a cursor position) by incorporating another person’s pre-recorded movement into the participant’s online movement. Accordingly, they could gradually perceive whether sensory feedback was attributed to self- or other control (self-other attribution). The second strategy was multivoxel pattern analysis (MVPA) of functional magnetic resonance imaging (fMRI) data (Haynes and Rees 2005; Kamitani and Tong 2005; Norman et al. 2006). MVPA makes it possible to explore neural information represented in distinct patterns of fMRI voxel signals (Haynes 2015; Hebart and Baker 2018). In the current study, we separately decoded self-other attribution and sensorimotor information (e.g., sensory prediction error), which was correlated with self-other attribution, and examined the preference for agency attribution (or for sensorimotor information) in the found regions. As a result, we found that the inferior parietal lobe (IPL), sensorimotor, anterior insula, and higher visual cortices contained the information that determined self-other attribution. Among the found regions, the right supramarginal gyrus (SMG) showed the highest preference for self-other attribution, compared with sensorimotor information, at the final stage of a movement. We acknowledge that the sense of agency is associated with not only sensorimotor information but also with external cues such as subliminal/supraliminal priming (Moore et al. 2009; Wenke et al. 2010; Chambon et al. 2013) or social context (Beyer et al. 2018), and that it is finally determined as a result of integrating multiple sources of information (Pacherie 2007; Moore and Fletcher 2012; Synofzik et al. 2013). Although multiple sources other than sensorimotor information also play a crucial role in the sense of agency, the current study focuses on the sense of agency grounded on the sensorimotor system illustrated in Figure 1.

## Materials and Methods

### Participants

Eighteen right-handed and healthy volunteers (six females) with a mean age of 25.9 years (20–42 years) participated in our experiment. Our previous study (Asai 2016), which adopted a similar experimental paradigm as a behavioral study, found a main effect of a morphing ratio condition on attribution judgment (for details of the experimental paradigm, see below). We calculated the sample size for our behavioral data because it is critical in the current study to obtain the effect of the morphing condition. We calculated this based on a power analysis for repeated measures analysis of variance (ANOVA) using G^*^power 3.1 with power selected at 0.8, effect size (*f*) at 0.4, and alpha at 0.05. According to the requirements of this analysis, sample size for behavioral data was nine participants. Eventually, we chose the sample size of this study to be 18 based on the estimated sample size for behavioral data and those used in previous fMRI studies of the sense of agency (David et al. 2007; Farrer et al. 2008; Nahab et al. 2011). Written informed consent was obtained from all of the volunteers in accordance with the latest version of the Declaration of Helsinki. The experimental protocol was approved by the ethics committee of Kochi University of Technology.

### Behavioral Task

#### Trial Timeline

**Figure 2:**
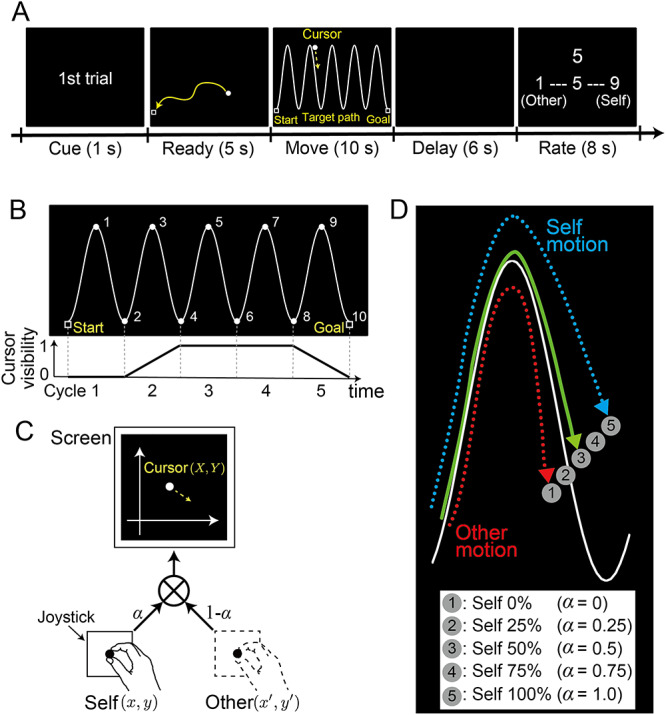
(*A*) Trial timeline. After moving a cursor to the start position (shown as a square) during the 5-s ready period, participants traced a sinusoidal target-path with a cursor controlled by a joystick during the 10-s move period. Following a 6-s delay period, participants assigned a score (on a 9-point Likert scale) to their self-other attribution by pushing buttons. (*B*) Target path (top). Numbers were sequentially presented every second to help participants maintain the required pace of tracing. Cursor visibility (bottom). The cursor was invisible on the screen during the first 2.0 s (first cycle). Visibility linearly increased from zero to one over the next 2.0 s (second cycle). Here, zero corresponds to black (RGB: 0, 0, 0), which is the same brightness as the background, while one corresponds to white (RGB: 255, 255, 255). The cursor continued to be clearly visible during the next 4.0 s (third and fourth cycles) and then linearly became darker from 8.0 to 10 s (fifth cycle). (*C*) Morphing method. Cursor position on the screen (*X*, *Y*) was the weighted summation of the joystick position controlled by the current participant (self) (*x*, *y*) and a pre-recorded joystick position (other) (*x*’, *y*’). Weights were modified by a morphing ratio ($\alpha$). (*D*) Cursor trajectories. Circles labeled with numbers (1–5) illustrate how the cursor position was changed according to the five morphing ratios ($0\le \alpha \le 1$): self 0% (number 1) to self 100% (number 5) at every 25% step. In the self-other mixed conditions (number 2, 3, or 4), the cursor was displayed between the position of the participant’s own joystick and the position of the other person’s joystick.

Participants were required to trace a five-cycle sinusoidal wave (target path) with a cursor (Fig. 2*A*) (Asai 2016). They manipulated a joystick with their right index finger to control the cursor on the screen.

*Cue and ready periods*. At the beginning of each trial, the trial number was displayed on the screen in a cue period of 1 s. While listening to four countdown sounds, participants set their cursor at the starting point located near the lower-left corner of the screen within a ready period of 5 s.

*Move period*. As soon as the target path was presented on the screen, participants started tracing it toward the goal point located near the lower-right corner of the screen. They were required to trace each half cycle of the target path in 1 s (i.e., 0.5 Hz) and to complete the entire movement within a move period of 10 s. We sequentially displayed numbers from 1 to 10 every second at the top and bottom of the sinusoidal target-path to help participants maintain the required pace of tracing (Fig. 2*B* top and Supplementary Movie 1). The cursor position on the screen was determined by the weighted summation of the participant’s online joystick and the pre-recorded other’s joystick position (for details see Morphing Visual Feedback from Self to Other). Each participant was instructed that the cursor movement on the screen corresponded to the participant’s own or to someone else’s joystick movement and that he or she should trace the target path with the cursor as accurately and as smoothly as possible. Note that the cursor was invisible on the screen during the first 2.0 s of the move period (first cycle) and then gradually appeared (i.e., brightness of the cursor linearly increased) during the next 2.0 s (second cycle). Furthermore, the cursor gradually disappeared (i.e., brightness of the cursor linearly decreased) from 8.0 to 10 s (fifth cycle) (Fig. 2*B* bottom and Supplementary Movie 1). The reason for this cursor-visibility control is that the onset and the offset of the cursor movement are sensitive to the mismatch between the participants’ own joystick and the cursor movements, which predominantly affects self-other attribution judgment (i.e., temporal delay and spatial deviation at the onset/offset timing might strongly affect judgment).

*Delay and rating periods*. A blank screen was presented during a 6-s delay period after the move period. Then participants reported how much they felt the cursor movement could be attributed to their own joystick movement on a 9-point Likert scale from 1 (completely the other’s movement) to 9 (completely their own movement). The number 5 was displayed on the screen at the beginning of a rating period for 8 s. The number was incremented or decremented by pressing the right or left button, respectively. The buttons were attached to the joystick box, and participants were instructed to press the buttons with their right hand.

#### Morphing Visual Feedback from Self to Other

Visual feedback (i.e., cursor movement) during the tracing movement was morphed by incorporating another person’s movement into the participant’s movement. We calculated the weighted summation of the participant’s online joystick position (*x*, *y*) and the other’s position (*x*’, *y*’) at 60 Hz (refresh rate of the monitor) and displayed the cursor in the calculated position (*X*, *Y*) on the screen (Fig. 2*C*). This weight corresponded to the morphing ratio ($\alpha$). The other persons’ movements were recorded prior to the fMRI experiment, and 240 trajectories (15 trajectories recorded from each of 16 participants) were stored in a dataset. A trajectory was randomly chosen for each trial from this dataset. Five morphing ratios were set at 25% intervals, from self 0% (other 100%, $\alpha =0$) to self 100% (other 0%, $\alpha =1.0$). In the self 100% condition, the visible cursor position fully corresponded to the participant’s joystick position (cursor labeled number 5 in Fig. 2*D*). By contrast, in the self 0% condition, the visible cursor position was independent of the participant’s own joystick position (cursor labeled number 1 in Fig. 2*D*). In the self-other mixed conditions, the cursor was displayed at a position between the position of the participant’s own joystick and that of the other person’s pre-recorded joystick (cursor labeled number 2, 3, or 4 in Fig. 2*D*).

#### Experimental Procedure

Before the main fMRI runs, participants performed two types of practice runs inside the fMRI scanner. In the first practice run, the participants were trained to trace the target path with a cursor moving in accordance with 1-Hz metronomic sounds to become accustomed to the cyclic movement. In this run, the cursor movement precisely reflected their joystick movement (self 100% condition). In the second practice run, they conducted the same task as the main fMRI runs but with a smaller number of trials (10 trials) than that of the main runs (50 trials/run). After the practice runs, participants conducted three main runs/day (150 trials) for a total of six runs (300 trials) over 2 days. The participants performed the task using the five morphing ratios 10 times in random order during each of the main runs.

### MRI Data Acquisition

A 3-T Magnetom Verio scanner (Siemens) with a 32-channel head coil was used to acquire T2^*^-weighted echo-planar images (EPI). In total, 753 volumes were acquired in each run with a gradient echo EPI sequence under the following scanning parameters: repetition time (TR), 2000 ms; echo time (TE), 30 ms; flip angle (FA), 70°; field of view (FOV), 192 × 192 mm; matrix, 64 × 64; 30 axial slices; and thickness, 4 mm with a 1-mm gap. T2-weighted turbo spin echo images were scanned to acquire high-resolution anatomical images of the same slices used for the EPI (TR, 6000 ms; TE, 58 ms; FA, 160°; FOV, 192 × 192 mm; matrix, 256 × 256; 30 axial slices; and thickness, 4 mm with a 1-mm gap). T1-weighted structure images were obtained with 1 × 1 × 1-mm resolution with a gradient echo sequence (repetition time, 2250 ms; echo time, 3.06 ms; flip angle, 9°; matrix, 256 × 256; 192 axial slices; and thickness, 1 mm without gap).

### Preprocessing of fMRI Data

The fMRI data were analyzed using SPM8 (Wellcome Trust Centre for Neuroimaging, London, UCL) on MATLAB. We discarded the first three volumes of the functional images in each run to allow for T1 equilibration. The remaining image volumes were temporally realigned to correct for the sequence of slice acquisition and then spatially realigned to the first image to adjust for motion-related artifacts. Rigid-body transformations were performed to align the functional images to the structural image for each subject. The images were spatially normalized with the Montreal Neurological Institute (MNI) (Montreal, Quebec, Canada) reference brain and resampled into 3 × 3 × 4-mm cuboid voxels. Note that spatial smoothing was not applied to the data, since this might blur the fine-grained information contained in multivoxel activity (Mur et al. 2009). After linear-trend removal within each run, we calculated the percentage of signal change relative to the mean of activity for each run.

### Decoding Self-Other Attribution with Multivoxel Pattern Regression

We performed multivoxel pattern regression to decode a self-other rating score (ranging from 1 to 9) from the fMRI activity patterns during movement. A linear support vector regression (SVR) model implemented in LIVSVM (http://www.csie.ntu.edu.tw/∼cjlin/libsvm/) was applied to the voxel patterns with the trial-by-trial rating score as a dependent variable. The SVR model was trained using the data from four out of five morphing conditions and then tested using the data in the remaining condition (i.e., leave-one-condition-out cross validation) to prevent the differences among conditions from becoming a confounding factor. In addition, we evaluated the decoding performance using a leave-one-run-out cross-validation procedure to prevent the differences among runs from becoming a confounding factor. More specifically, we trained the model with the fMRI data from four out of five conditions in five out of six runs and tested it with the independent data of the remaining condition in the remaining run (e.g., the fifth condition in the sixth run). This procedure was repeated 30 times (five conditions times six runs) so that each condition in each run was used as test data once. We performed a volume-based searchlight analysis using voxels within a 9-mm-radius sphere (see Searchlight Decoding Over the Brain) extracted from each volume of fMRI data scanned every 2 s (TR = 2 s) during the move periods. Here, each volume corresponded to a cycle of the sinusoidal movement.

#### Evaluation of Decoding Accuracy in Individual Analyses

We evaluated the above decoding accuracy in the test phase by calculating the *z*-scores of the Fisher-transformed Pearson’s correlation coefficient following the permutation procedure (Langfelder et al. 2011; Shibata et al. 2016). We generated 1000 surrogate correlation coefficients by permutating the relationship between the actual and predicted value 1000 times to get an empirical distribution of the correlation coefficients. The *z*-scores of the original (without permutation) value was calculated based on the empirical distribution. The above steps were then applied to the test dataset in each condition. We regarded *z*-scores averaged across conditions as indicative of the decoding performance by each participant.

#### Searchlight Decoding Over the Brain

We performed a volume-based searchlight decoding analysis (Kriegeskorte et al. 2006; Haynes et al. 2007). We repeatedly extracted voxel patterns within a 9-mm-radius sphere containing at least 65 voxels to perform regression analysis. This sphere was moved over the gray matter of the entire brain, and the mean of the *z*-scores was assigned to the sphere’s central voxel, resulting in a 3-D *z*-score map for each participant. A random-effects group analysis was performed on the *z*-score maps by using SPM8. To satisfy the assumptions of Gaussian random field theory for statistical inference at the group level, the *z*-score maps were smoothed with a 4-mm full-width at half-maximum (FWHM) Gaussian kernel (Soon et al. 2008; Bode and Haynes 2009; Wisniewski et al. 2015, 2016). We applied a statistical analysis of the entire brain with a threshold of *P* < 0.01 (family-wise error (FWE) corrected at cluster level with a cluster-forming threshold of *P* < 0.0005). The anatomical localization was determined according to the automated anatomical labeling (AAL) atlas (Tzourio-Mazoyer et al. 2002).

### Decoding Cursor-Joystick Distance and Velocity Difference with Multivoxel Pattern Regression

We performed multivoxel pattern regression to investigate whether the brain regions contained enough information to predict sensorimotor information (distance or velocity difference between the cursor and joystick; for details and definition, see Results: Relationship Between Tracing Behavior and Rating Score of Self-Other Attribution). Note that we used the mean of the distance or velocity difference from 4 to 8 s after the onset of the move period in order to exclude periods when the cursor was not clearly visible on the screen (for details see Behavioral Task). The procedure for decoding sensorimotor information was almost the same as that for decoding self-other attribution. The only exception was that the dataset in the self 100% condition was not included in the calculation of decoding performance (i.e., a correlation coefficient between actual and predicted value) because the cursor-joystick distance or velocity difference was zero in this condition. We applied a volume-based searchlight analysis to each volume of fMRI data scanned every 2 s during the move period. We assigned *z*-scores of the Fisher-transformed Pearson’s correlation coefficient calculated by the permutation procedure (see Evaluation of Decoding Accuracy in Individual Analysis) to the sphere’s central voxel. Next, we performed group analysis on the smoothed 3-D *z*-score maps for each participant.

## Results

### Self-Other Rating Score on Morphing Ratio Condition

**Figure 3:**
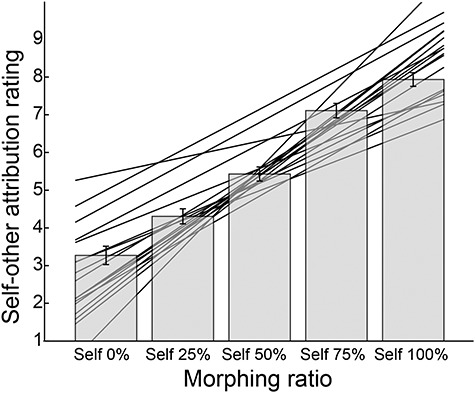
Self-other attribution rating scores averaged across participants for each morphing ratio. The higher the score was, the more strongly participants felt that the cursor movement was attributed to their own joystick movement. Error bars indicate standard error of the mean. A linear regression model was fitted to each participant’s rating scores. The regression lines of all participants are shown behind the bars.

Figure 3 shows the rating score averaged across all participants as a function of the morphing (self-movement) ratio. We found a significant main effect of the morphing ratio (*F* (4, 85) = 151.2, *P* < 0.001) according to a one-way repeated-measures ANOVA with the five morphing ratios as a within-subject factor. The rating scores linearly increased as the ratio of self-movement increased, which was also found in a previous study (Asai 2016). The lines behind bars represent regression lines fitted to each participant’s rating scores: (rating score) = *w*_0_ + *w*_1_ × (self-movement ratio). The mean of the line slopes (*w*_1_) was 0.049 (SD: 0.015), which was significantly larger than zero (two-tailed *t*-test: *t*(17) = 13.6, *P* < 0.001).

### Relationship Between Tracing Behavior and Rating Score of Self-Other Attribution

**Figure 4:**
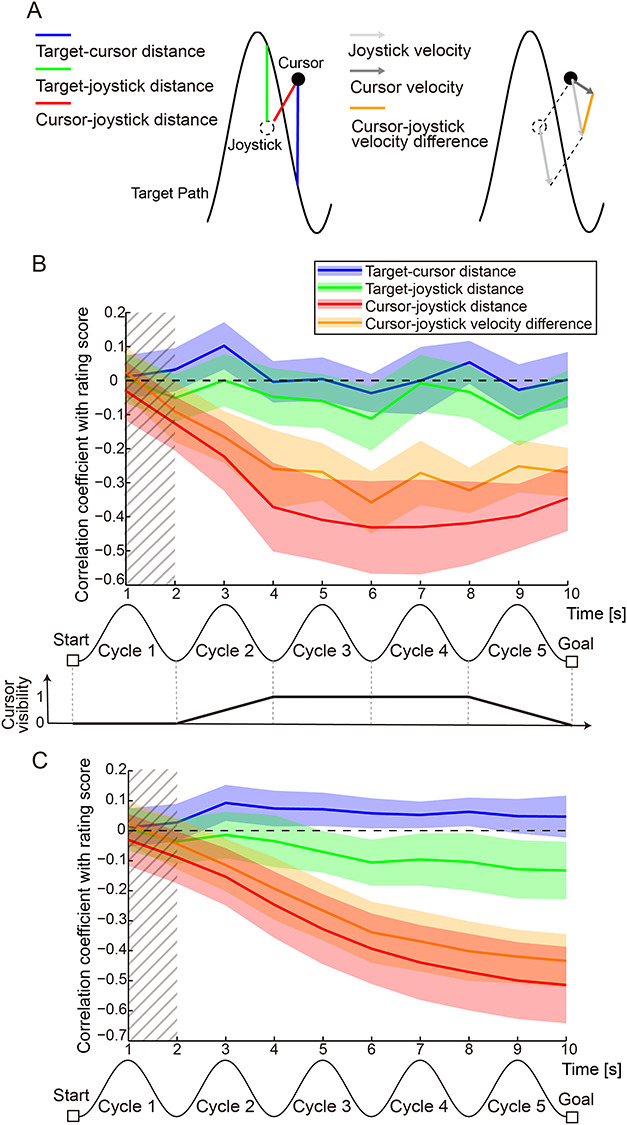
Relationship between tracing behavior and rating score of self-other attribution of movement. (*A*) Schematic of the four behavioral measures whose relationships with the self-other attribution score were investigated. Blue, green and red lines in the left panel indicate the target-cursor, target-joystick, and cursor-joystick distances, respectively. Light and dark gray arrows in the right panel denote the joystick and cursor velocity, respectively. Orange line represents the cursor-joystick velocity difference. (*B*) Time courses of Fisher-transformed Pearson’s correlation coefficients between each behavioral measure and the self-other rating scores during the 10-s move period. Values of behavioral measures were averaged within every second. Colored shaded areas denote 95% confidence intervals. Bottom panel denotes visibility of the cursor during the move period. Hatched area denotes the period during which the cursor was invisible (i.e., cursor visibility was zero). Here, the data for self 50% condition are shown. Negative correlation indicates that the greater the behavioral measure became, the lower the score the participants gave (i.e., more other attribution). (*C*) Time courses of Fisher-transformed Pearson’s correlation coefficients between accumulated value of each behavioral measure and rating scores. Values of behavioral measures were averaged from movement onset to every second. Colored shaded areas denote 95% confidence intervals. Hatched area denotes the period during which the cursor was invisible. Note that the data for self 50% condition are shown. Negative correlation indicates that the greater the behavioral measure was accumulated, the lower the score the participants gave.

We investigated the relationship between trial-by-trial rating scores and behavioral measures to specify the sensorimotor factors that affected the self-other attribution of the participants. We examined four behavioral measures as possible factors: 1) the target-cursor distance, which is the vertical distance between the target path and the cursor position (blue line in left panel of Fig. 4*A*), 2) the target-joystick distance, which is the vertical distance between the target path and the joystick position (green line in left panel of Fig. 4*A*), 3) the cursor-joystick distance, which is the Euclidean distance between the cursor and the joystick positions (red line in left panel of Fig. 4*A*), and 4) the cursor-joystick velocity difference. The velocity difference was the norm of the difference between the cursor and the joystick velocities: $\sqrt{{({v}_{xc}-{v}_{xj})}^2+{({v}_{yc}-{v}_{yj})}^2},$ where ${v}_{xc}$ and ${v}_{yc}$ are *x*- and *y*-direction velocities of the cursor, respectively, and ${v}_{xj}$ and ${v}_{yj}$ are those of the joystick, respectively (orange line in right panel of Fig. 4*A*). We calculated the Fisher-transformed Pearson’s correlation coefficients between each behavioral measure (mean value within every second) and self-other rating scores (one value for each trial).

**Figure 5:**
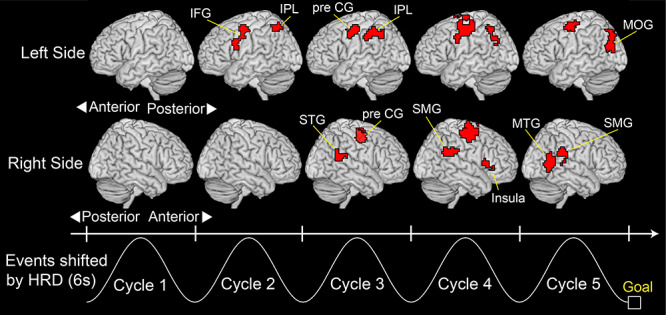
Decoding performance for self-other attribution during movement, with clusters of significant decoding accuracy (*P* < 0.01 FWE corrected at cluster level with a cluster-forming threshold of *P* < 0.0005). A searchlight decoding analysis was applied to a volume scanned every 2 s during the 10-s move period to create accuracy maps. The sinusoidal waves represent a typical cursor movement along the timeline shifted by 6 s from the actual time considering the hemodynamic response delay (HRD). All clusters larger than 50 voxels are reported. IFG: inferior frontal gyrus, IPL: inferior parietal lobe, pre CG: precentral gyrus, MOG: middle occipital gyrus, MTG: middle temporal gyrus, SMG: supramarginal gyrus, STG: superior temporal gyrus.

**Table 1:** Summary of searchlight decoding of self-other attribution of movement during move period

Brain region	Side	Cluster size	MNI coordinates (peak voxel)		
			*x*	*y*	*z*
Cycle 2					
1. Inferior frontal gyrus	Left	115	−51	11	26
2. Inferior parietal lobe	Left	52	−39	−58	54
Cycle 3					
3. Inferior parietal lobe	Left	147	−42	−52	38
4. Precentral gyrus	Left	198	−39	−1	38
5. Precentral gyrus	Right	193	36	−10	50
6. Superior temporal gyrus	Right	56	57	−43	18
Cycle 4					
7. Precentral gyrus	Left	367	−45	−13	50
8. Inferior parietal lobe	Left	81	−39	−58	50
9. Precentral gyrus	Right	339	33	−10	58
10. Insula	Right	79	33	17	6
11. Supramarginal gyrus	Right	77	57	−46	26
Cycle 5					
12. Precentral gyrus	Left	82	−36	−10	54
13. Middle occipital gyrus	Left	150	−36	−73	22
14. Middle temporal gyrus	Right	84	54	−58	14
15. Supramarginal gyrus	Right	50	60	−34	30

*Note*: A threshold at *P* < 0.05 (FWE-corrected at cluster level with a cluster-forming threshold of *P* < 0.0005) was set for statistical testing. Clusters larger than 50 voxels are reported. Cycles correspond to those illustrated at the bottom of Figure 5, and they are shifted by 6 s from the actual time considering the HRD.

**Figure 6:**
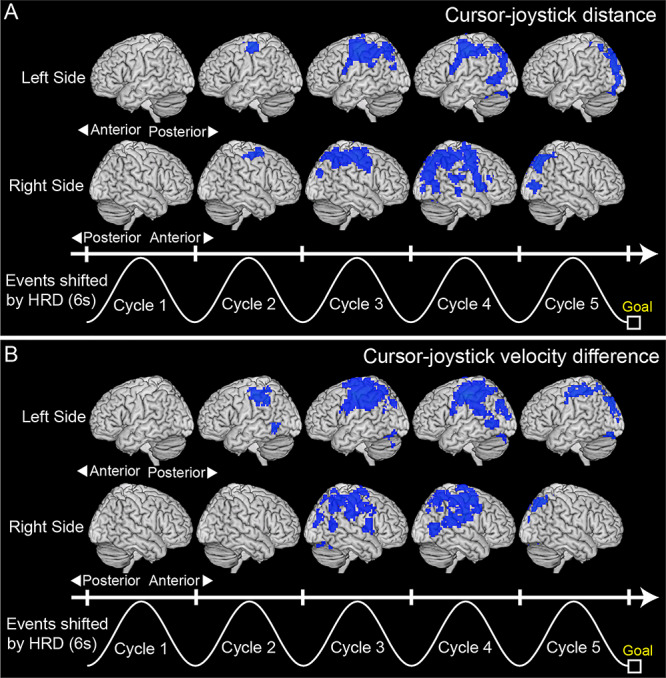
(*A*) Clusters showing significant decoding accuracy for cursor-joystick distance and (*B*) those for cursor-joystick velocity difference (blue regions; *P* < 0.01 FWE-corrected at cluster level with a cluster-forming threshold of *P* < 0.0005). A searchlight decoding analysis (Kriegeskorte et al. 2006) was applied to a volume scanned every 2 s during the 10-s move period to create accuracy maps. The sinusoidal waves represent a typical cursor movement along the timeline shifted by 6 s from the actual time considering the HRD. All clusters larger than 50 voxels are reported.

Figure 4*B* shows the time course of the correlation for the self 50% condition (Supplementary Fig. 1 shows time courses for all conditions). In the self 50% condition, the upper limit of the 95% confidence interval (CI) of the cursor-joystick distance was lower than zero from 2 s after the onset of movement (red line in Fig. 4*B*). Similarly, the 95% CI of the velocity difference was less than zero from 3 s after the onset of movement (orange line in Fig. 4*B*). This negative correlation indicates that the greater the cursor-joystick distance or velocity difference was, the more likely were the participants to judge the cursor movements to be attributed to the other’s motion, and vice versa.

As shown in the bottom panel of Figure 4*B*, we controlled the cursor visibility to avoid participants’ highly sensitive reaction to the initial and final mismatch between their joystick and the cursor movements. Since the cursor visibility was different among the periods between 0 and 2 s (first cycle), between 2 and 4 s (second cycle), between 4 and 8 s (third and fourth cycles), and between 8 and 10 s (fifth cycle), the correlation coefficients for the four periods were not considered comparable. The correlations were nearly zero between any of the behavioral measures and the rating score in the first cycle. This result is reasonable because the cursor was completely invisible in this period. The cursor-joystick distance and velocity difference were highly correlated with each other: Pearson’s correlation coefficients averaged across participants were 0.85 (SD: 0.10), 0.83 (0.10), 0.72 (0.17), and 0.38 (0.22) in the self 75%, 50%, 25%, and 0% conditions, respectively. Note that we used the mean of the distance or velocity difference from 4 to 8 s after the onset of the move period. This high correlation seems reasonable because these measures are not independent but determined by the relationship between the cursor and joystick movements, which largely affected the rating score.

Figure 4*C* shows the time courses of the correlation between the rating score and the accumulated value of each measure, which was averaged from movement onset to each second. The negative correlation gradually became larger according to the distance or velocity-difference accumulation (red and orange lines in Fig. 4*C* shown for self 50% condition). The time courses for all conditions are shown in Supplementary Fig. 2. This finding indicates that the accumulation of distance or velocity difference between cursor and joystick is essential for the judgment of self-other attribution. By contrast, the correlation coefficients for the target-cursor and target-joystick distances were stable around zero (blue and green lines in Fig. 4*B*,*C*).

### Decoding Self-Other Attribution During Movement

We decoded self-other attribution of cursor movement, which was evaluated by the participants after movement, from fMRI voxel patterns during the tracing. A searchlight analysis found clusters in which self-other attribution could be significantly decoded from their voxel patterns (red regions in Fig. 5, *P* < 0.01 FWE-corrected at cluster level with a cluster-forming threshold of *P* < 0.0005; all clusters are reported in Table 1). At first, the clusters in the left inferior frontal gyrus and IPL appeared in the second cycle of the move period. Then, the bilateral precentral gyrus, right superior temporal gyrus, right IPL (mainly the SMG), and right anterior insula showed significant decoding accuracies from the third to fourth cycles. In the last cycle, the left middle occipital gyrus and right middle temporal gyrus showed significant accuracies. These results indicate that the information that could predict the following self-other attribution is contained in the regions reported as the neural correlates of the sense of agency by the previous studies, such as the posterior parietal (Farrer and Frith 2002; Farrer et al. 2003, 2008; Ogawa and Inui 2007; Schnell et al. 2007; Yomogida et al. 2010; Nahab et al. 2011), sensorimotor (David et al. 2008; Sperduti et al. 2011), anterior insula (Farrer et al. 2003; Tsakiris et al. 2010), and higher visual cortices (Astafiev et al. 2004; David et al. 2007; Yomogida et al. 2010). We also showed that the neural representation shifted from region to region during the tracing.

When assessing an individual’s self-other discriminability by fitting the linear regression model to each participant’s rating scores (regression lines in Fig. 3), we found some of the participants showing relatively low and unstable discriminability. Therefore, as an additional analysis, we examined whether the poor discriminability affected our decoding result. We excluded seven (out of 18) participants with relatively low and unstable discriminability according to certain criteria (see Supplementary Results) and performed a random-effects group analysis on the *z*-score maps of the remaining participants. Consequently, we found a result similar to that of the 18 participants (for details see Supplementary Results: Exclusion of participants with low and unstable discriminability of action attribution and Supplementary Fig. 3).

### Preference for Self-Other Attribution or for Sensorimotor Information

We next decoded the sensorimotor information that was correlated with self-other attribution (i.e., cursor-joystick distance and velocity difference, Fig. 4) from fMRI voxel patterns using a searchlight analysis. As a result, we were able to decode the cursor-joystick distance in many regions (Fig. 6*A*, *P* < 0.01 FWE-corrected at cluster level with a cluster-forming threshold of *P* < 0.0005), including some of the clusters shown in Figure 5. Similar results were obtained for the velocity difference (Fig. 6*B*). Note that we could not find any cluster showing significant decoding performance in the first cycle. This is reasonable because the cursor was not displayed for the first 2 s (Fig. 2*B*). These results indicate the possibility that decoding performance in some clusters in Figure 5 more dominantly reflected sensorimotor information than self-other attribution.

We assessed whether the voxel patterns in each cluster in Figure 5 were more sensitive to self-other attribution than to sensorimotor information or vice versa as follows. We computed difference (*diff*) in decoding performance (measured by *z*-score, see “Evaluation of Decoding Accuracy in Individual Analysis” in Materials and Methods) between self-other attribution and sensorimotor information. Here, *diff* was calculated for each participant (*i* = 1, 2, … 18) and each cluster (*j* = 1, 2, … 15):

(1) \begin{eqnarray*} \mathrm{diff}\left(i,j\right) &=& z\hbox{-} {\mathrm{score}}^{\mathrm{self}\hbox{-} \mathrm{other}\ \mathrm{attribution}} \left(i,j\right)\nonumber\\ & -& z\hbox{-} {\mathrm{score}}^{\mathrm{sensorimotor}\ \mathrm{information}}\left(i,j\right). \end{eqnarray*}

As mentioned above, the cursor-joystick distance highly correlated with velocity difference. Thus, we compared the mean *z*-score across participants between the two measures for each cluster and chose the higher one as *z*-score^sensorimotor information^ for the cluster (*j*) to simplify further analysis. Note that the mean *z*-score was higher for the cursor-joystick distance than for the velocity difference in all clusters except cluster 15 (right SMG, see Table 1). We computed mean *diff* across participants for each cluster (*M*_*diff*(*j*)_) and divided this by the standard deviation (SD_*diff*(*j*)_) to get the effect size (Cohen’s *d_z_*, Lakens 2013):

(2) \begin{equation*} \mathrm{Cohen}^{\prime}\mathrm{s}\ {d}_z(j)=\raisebox{1ex}{${M}_{diff(j)}$}\!\left/ \!\raisebox{-1ex}{${\mathrm{SD}}_{diff(j)}$}\right., \end{equation*}

where ${\mathrm{SD}}_{diff(j)}=\sqrt{\frac{\sum_{i=1}^{18}{( diff(i,j)-{M}_{diff(j)})}^2}{18-1}}$. Importantly, this effect size has a sign. The positive sign means that the cluster (*j*) is more sensitive to self-other attribution than to sensorimotor information, while the negative sign means the opposite case (see eq. 1).

**Figure 7:**
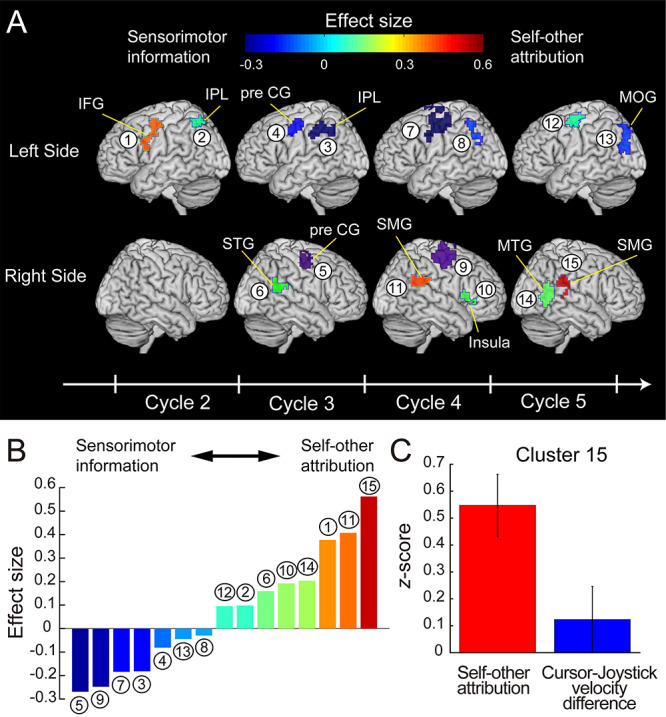
(*A*) Clusters color-coded according to the effect size of the difference between decoding performance for self-other attribution and for sensorimotor information. Warmer colors denote bias toward self-other attribution (positive value of the effect size), while colder colors denote bias toward sensorimotor information (negative value). The clusters are identical to those in Figure 5 (see Table 1 for anatomical details). The sinusoidal wave represented at the bottom is shifted by 6 s from the actual time considering the HRD. (*B*) Effect sizes in the 15 clusters sorted in ascending order. The number and color of the bars correspond to those of clusters in (*A*). (*C*) Decoding performance (*z*-score) for self-other attribution and cursor-joystick velocity difference in the right SMG in the fifth cycle (red and blue bars, respectively). Error bars show standard error of the mean.

Figure 7*A* represents the signed effect sizes of the 15 clusters using a color code. Reddish colors indicate a bias toward the self-other attribution (positive sign), while bluish colors indicate a bias toward the sensorimotor information (negative sign). Figure 7*B* shows the effect sizes of the clusters sorted in ascending order (see Supplementary Fig. 4 for decoding performances for self-other attribution and sensorimotor information in all clusters). According to Figure 7*B*, the areas near the central sulcus, including the bilateral precentral gyrus (clusters 5, 7, and 9) and left IPL (cluster 3), showed prominent biases toward sensorimotor information. By contrast, effect sizes in the right SMG in the fourth and fifth cycles (clusters 11 and 15) showed the highest and second-highest values among the clusters, respectively. Consistent with this result, the decoding performance in the right SMG was significantly higher for self-other attribution than for sensorimotor information (cursor-joystick velocity difference) in the fifth cycle (paired *t*-test: *t*(17) = 2.38, *P* = 0.029, Fig. 7*C*). In addition, we found the left IFG in the second cycle (cluster 1), right MTG in the fifth cycle (cluster 14), right anterior insula in the fourth cycle (cluster 10), and right STG in the third cycle (cluster 6) to show relatively high effect sizes. Note that sensorimotor information was not considered comparable among the periods between 2 and 4 s (second cycle), between 4 and 8 s (third and fourth cycles), and between 8 and 10 s (fifth cycle) since we controlled the cursor visibility (for details see Materials and Methods: Behavioral task). Taken together, our results reveal that the preference for self-other attribution (or sensorimotor information) was different among the 15 clusters. Notably, the right SMG is the most sensitive to self-other attribution among these clusters at the final stage of movement.

**Figure 8:**
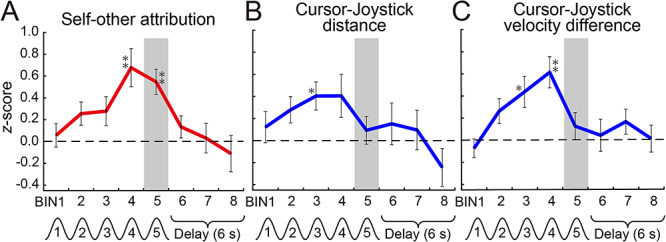
Time courses of *z*-scores for self-other attribution (*A*), cursor-joystick distance (*B*), and velocity difference (*C*) at the peak voxel (*x* = 60, *y* = −34, *z* = 30 in MNI coordinates) in the right SMG (cluster no. 15 in Fig. 7). Error bars show standard error of the mean. Asterisks indicate *z*-scores that were significantly larger than zero according to two-tailed one-sample *t*-test (^*^: *P* < 0.01 uncorrected, ^*^^*^: *P* < 0.05 Bonferroni corrected for multiple comparisons). Each time bin corresponds to a volume scanned every 2 s during the 10-s move and 6-s delay periods. Gray bars highlight the fifth bin (last cycle of the sinusoidal movement). The events denoted under the time bins are shifted by 6 s from the actual time considering the HRD.

We further investigated the temporal changes in decoding performances (*z*-scores) of the cluster in the right SMG. Figure 8 shows *z*-scores as a function of the time bin (2 s) for self-other attribution (Fig. 8*A*), cursor-joystick distance (Fig. 8*B*), and velocity difference (Fig. 8*C*) at the peak coordinate (*x* = 60, *y* = −34, *z* = 30 in MNI coordinates). The *z*-score for self-other attribution reached the peak value in the fourth cycle (time bin 4) and maintained a significant value in the last cycle (time bin 5: gray bars). Meanwhile, the *z*-score for sensorimotor information reached the peak before the last cycle (third cycle for cursor-joystick distance and fourth cycle for velocity difference) but abruptly declined in the last cycle. Consequently, while the decoding performance for sensorimotor information reached a significant level during the middle stage of the move period, the performance for self-other attribution remained high at the end of the move period.

## Discussion

In the current study, we first found the sensorimotor, posterior parietal, anterior insula, and higher visual cortices as the regions where the self-other attribution could be decoded from their voxel patterns (Fig. 5). As we found a tight relationship between agency attribution and the sensorimotor information based on the correspondence between the cursor and joystick movement (cursor-joystick distance and velocity difference; Fig. 4), some of the found regions overlapped those in which the sensorimotor information could be decoded. Then, we investigated whether information represented in the found regions showed a preference for self-other attribution or for sensorimotor information according to the effect size of the difference in decoding performance. As a result, the right SMG (at the late stage of movement) and left IFG (at the early stage of movement) were sensitive to self-other attribution, while the bilateral precentral gyri and left IPL dominantly reflected sensorimotor information (Fig. 7). Our findings demonstrate that individual regions processed the different levels of information during movement according to their preference.

The comparator model proposed that the prediction error is the main factor to determine agency attribution (Blakemore et al. 2000; Frith et al. 2000). In our experiment, the joystick position was not displayed on the screen during the task. However, participants could predict the actual position and velocity of their joystick on the screen according to their proprioception and a forward model of the relationship between the cursor and joystick. The participants could acquire the forward model during the practice run under the self 100% condition (for details see Materials and Methods: Behavioral Task). A cursor appeared in the position shifted by the addition of the other’s joystick position to the predicted position (i.e., actual position) in the self 0–75% conditions (Fig. 2*D*). Therefore, we can regard the cursor-joystick distance and velocity difference (Fig. 4*A*) as proxies for a prediction error of sensory feedback. Our results demonstrate that the accumulation of these behavioral measures explains a large part of the variance in self-other attribution (red and orange lines in Fig. 4*B*,*C*). These results are consistent with the implications suggested by the comparator model.

Previous studies have reported multiple brain regions as neural bases for the sense of agency. We found many such regions to be those in which self-other attribution could be decoded from their voxel patterns, such as the posterior parietal (Farrer and Frith 2002; Farrer et al. 2003, 2008; Ogawa and Inui 2007; Schnell et al. 2007; Yomogida et al. 2010; Nahab et al. 2011), sensorimotor (David et al. 2008; Sperduti et al. 2011), anterior insula (Farrer et al. 2003; Tsakiris et al. 2010), and higher visual cortices (Astafiev et al. 2004; David et al. 2007; Yomogida et al. 2010). Since the concept of the sense of agency covers multiple aspects from the sensorimotor to judgment level, previous studies have not necessarily shown a difference in the level of the process in which each of various regions was involved within a single experiment (but see Farrer et al. 2008; Miele et al. 2011 for dissociation of brain regions associated with agency attribution from sensorimotor stages). The current study questioned whether the found regions preferentially processed immediate output of sensorimotor processing (mainly sensory prediction error) or information closely related to a conceptual judgment of agency during movement. We answered the question by showing the gradual difference in preference for self-other attribution in contrast to sensorimotor information (Fig. 7). Such gradation of neural representation would give us a clue to understanding how each region intermediates between the prediction error and the conceptual judgment of agency attribution.

We found the clusters in the right SMG at the late stage and left IFG at the early stage of movement as the top three clusters that were more sensitive to agency attribution than to sensorimotor information among the 15 clusters in which self-other attribution could be decoded (Fig. 7). The SMG is a part of the IPL, and previous studies also suggested that the right IPL plays a critical role in the subjective experience of self-agency. First, the right IPL is the region most frequently reported as a neural correlate of the sense of agency (Farrer and Frith 2002; Farrer et al. 2003, 2008; Ogawa and Inui 2007; Schnell et al. 2007; Nahab et al. 2011). In particular, Nahab et al. (2011) morphed visual feedback (finger movement) by incorporating pre-recorded movement into the participant’s actual one, and found two distinct networks involving the sense of agency: the leading and lagging networks. They found that blood-oxygen-level-dependent responses in the lagging network regions were slightly later than those in the leading network regions. By adding the functional connectivity analysis to this finding, they suggested that the lagging network played a role in mediating sensorimotor information to a conscious awareness of self-agency. The right IPL was found to be one of the main components of the lagging network. Second, a single-pulse transcranial magnetic stimulation (TMS) over the right IPL induced a change in the participant’s action attribution (Preston and Newport 2008; Ritterband-Rosenbaum et al. 2014; Chambon et al. 2015). Finally, the right IPL reflects the subjective experience of self-agency even without receiving a prediction-error signal calculated in the sensorimotor system (Wenke et al. 2010; Haggard and Chambon 2012; Chambon et al. 2013, 2014, 2015; Haggard 2017; Beyer et al. 2018). The previous studies reported that the prospective signals, particularly the fluency of action selection controlled by subliminal priming, affected the sense of agency (Wenke et al. 2010). Their fMRI studies suggested that a region in the right IPL received the prospective signals carried from the dorsolateral prefrontal cortex and constructed the subjective experience of self-agency (Chambon et al. 2013). Taken together, the previous findings support our conclusion that the right IPL is dominantly responsible for the higher-order function in the neural process of the sense of agency (Eddy 2016), compared with the low-level sensorimotor component.

Regarding the role of the right IPL, many neuroimaging studies have reported the right angular gyrus (AG), not the right SMG, to be sensitive to the difference in the sense of agency (Farrer et al. 2008; Chambon et al. 2013; Beyer et al. 2018; but see Koreki et al. 2019). In the current study, we found the cluster mostly in the right SMG in the last cycle (cluster 15 in Fig. 7*A*), which did not overlap the right AG defined in the AAL atlas (Supplementary Fig. 5). There are two possible interpretations why the cluster was found in the right SMG, but not the right AG, in the current study. The first is that the SMG may preferentially code for sensorimotor conflicts, which are critical for the sense of agency, compared with an intersensory conflict between vision and proprioceptive information, which affects both sense of agency and body ownership (Tsakiris et al. 2010). Tsakiris et al. (2010) found the most prominent activation in the right SMG when visual feedback was asynchronized with the active finger movements. In contrast, the right AG was activated when visual feedback was asynchronized with passive movements as well as active movements. Based on their findings, it is possible to interpret that the neural representation in the right SMG found in our study reflected the sensorimotor-based sense of agency, not that affected by gain/loss of body ownership. The second is due to the difference in the analysis method between the previous studies and our study (i.e., univariate vs. multivariate analysis). To our knowledge, all of the previous neuroimaging studies reporting the AG as the neural basis of the sense of agency performed univariate analysis (i.e., increase or decrease in voxel-wise activation). By contrast, we performed MVPA to find the brain regions where we could decode agency attribution. Thus, it might be possible that the SMG mainly represents information reflecting the sense of agency at a multivoxel pattern level.

Our findings on the time courses of decoding performances in the right SMG (Fig. 8) have significant implications for the neural process of the sense of agency. The sensorimotor information could be decoded in the middle of the move period (third and fourth cycles in Fig. 8*B*,*C*). But more importantly, we found that the decoding performance for self-other attribution remained at a significant level (fifth cycle in Fig. 8*A*), despite the sudden decline in performance for sensorimotor information at the end of the move period (fifth cycle in Fig. 8*B*,*C*). The results suggest that the right SMG possibly contained the sensorimotor information for translating it into the conscious experience of self-agency. The right IPL has connections with many brain regions (Bzdok et al. 2013) and the role of multisensory integration (Ionta et al. 2011; Jakobs et al. 2012). Therefore, we can hypothesize that the right SMG receives and integrates the information processed in the regions that have a preference for sensorimotor processing and then calculates the sense of agency. For validation of this hypothesis, we need further studies on the information flow between the right SMG and other sensorimotor regions (Koreki et al. 2019).

As well as the right SMG, the left IFG in the second cycle (cluster 1 in Fig. 7*A*) also showed relatively high effect size among 15 clusters. The cluster on the left IFG in the second cycle largely overlaps those found in the previous studies on body ownership and peripersonal space (Ehrsson et al. 2004, 2005; Petkova et al. 2011; Gentile et al. 2013; Guterstam et al. 2013, 2015; Blanke et al. 2015; Grivaz et al. 2017). In the second cycle, we manipulated the cursor to gradually become visible as noted in the Materials and Methods section (for details see Behavioral Task section, Fig. 2*B* and Supplementary Movie 1). At the initial stage of a trial in this task, a possible strategy for participants was to explore the correspondence between their joystick control and the gradually visible cursor. Thus, we assumed that the degree of this correspondence determined how much the boundary of peripersonal space extended to the cursor on the screen. Bassolino et al. (2010) has already demonstrated that the peripersonal space around the hand can be extended toward a cursor (computer mouse) on the screen. This subjective feeling of extending their body to the cursor could affect their evaluation of agency attribution. The above interpretation is also supported by the fact that the other cluster in the second cycle (cluster 2 in Fig. 7*A*), which is located on the left IPL, also overlaps the area associated with body ownership and peripersonal space.

We combined two strategies to test our hypothesis. The first was to use a unique experimental paradigm that considers the temporal evolution of self-agency (Asai 2015, 2016, 2017). Most previous studies required participants to perform an intermittent action such as a button press (e.g., Farrer and Frith 2002) or a reaching movement (e.g., David et al. 2007). However, such simple tasks made it difficult to shed light on the processes of how the sense of agency was built in the brain. In our experiment, participants continuously received sensorimotor evidence while tracing a target path under an ambiguous condition so that they could gradually realize whether a cursor movement was attributed to the self or other. Thus, our task paradigm was appropriate to investigate the neural representation reflecting self-other attribution, which shifts from time to time. The second strategy was to apply an MVPA to fMRI data (Haynes and Rees 2005; Kamitani and Tong 2005; Norman et al. 2006). The MVPA enabled us to find the regions whose voxel patterns were more sensitive to self-other attribution than to sensorimotor information. Thus, the combination of these strategies has revealed a temporal change in the neural representation of self-agency grounded on the sensorimotor system. Note that MVPA evaluates whether the nonuniform response of voxels in a region is informative about the variable of interest (Hebart and Baker 2018). Therefore, it is basically impossible to discuss how the uniform (i.e., positive or negative) responses in the found clusters were related to self- or other agency. Related to the above, we have confirmed that it is unlikely that our MVPA result (Fig. 5) could be explained only by the activation level in the found clusters (for more details see Supplementary Results: Mass univariate analysis of voxel-wise activation modulated by self-other attribution and Supplementary Fig. 6).

In the current study, participants were required to report self-other attribution of cursor movement as a judgment of agency. By contrast, some studies required participants to report different types of agency judgments such as those regarding controllability (i.e., how much control they felt). The current experiment was not designed to answer the question of whether the type of agency judgment influences the neural process for determining the agency judgment. To answer this question, we need future studies designed to reveal the difference in neural substrates implicated in different types of agency judgments. For instance, we will acquire fMRI data under two conditions as follows. In one condition, participants will be required to report their explicit judgment about “self-other attribution” (i.e., how much they felt that the cursor movement was attributed to their own action). In the other condition, participants will report their judgment of “controllability” (i.e., how much they felt that they could control the cursor). For MVPA, a decoder will be trained to predict a rating score of self-other attribution in the same way as the current study. In the test phase, we will evaluate whether the trained decoder can predict the rating score of controllability (i.e., cross-decoding). This study might shed light on whether a common brain region is recruited in different types of agency judgment.

The MVPA in our study has several possible confounding factors. The first factor is the difference in attention level used to control the cursor depending on whether the participants felt the cursor movement was attributed to the self or controlled by the other. A possible scenario could be as follows: Participants might find it more difficult to precisely control the cursor in the presence of an external agent controlling the cursor. This scenario would suggest that the more strongly the participants felt that an external agent controlled the cursor, the more attention they would have given toward controlling the cursor. In that case, we could have only decoded different levels of attention that covary with action attribution. We checked whether the attention level correlated with the rating score of the self-other attribution. Although we cannot directly measure the level of attention given to cursor control, it can be inferred from the accuracy of the participant’s tracing performance, such as the error between the target path and the cursor position (target-cursor distance in Fig. 4*A*). Note that we instructed participants to precisely trace the target path with the cursor even in the self-other mixed conditions. We found that the target-cursor distance did not correlate with the rating score (blue line in Fig. 4*B*). This result suggests that the attention placed on cursor control was not a crucial factor in decoding the self-other attribution judgment. The second factor is the difference in the rating score, which participants prepared in their minds before the rate period. We instructed them to judge action attribution on a 9-point Likert scale. Although the rate period was temporally distinct from the move periods, participants might have kept a rating score in mind before the rate period. It has been suggested that the right IPL is involved in a magnitude system of numerical processing (Walsh 2003). Thus, we might have decoded the difference in the rating score from activity patterns in the right SMG, even before the rate period. However, the decoding performance of the cluster in the right SMG declined once the delay period began (time bin 6 in the “self-other attribution” panel in Fig. 8*A*). This decline suggests that numerical processing was not a crucial factor in our successful regression.

In 1890, William James proposed the concept of the “I” as one aspect of the self: experiencing oneself as a subjective agent of thought, perception, and action (James 1890). Our study tackled the neural substrate underlying the awareness of ourselves as agents of action through interaction with the external world. As emphasized in the comparator model, our findings support the idea that the sense of agency is grounded on the sensorimotor system. More importantly, our study demonstrated the neural process that bridges the gap between lower level sensorimotor processing and higher level processing for agency attribution. In this process, the right SMG plays a critical role in translating sensorimotor information (obtained from interaction with the external world) into an awareness of the subjective agent of an action.

## Supplementary Material

Supplementary_bhaa028

Supplementary_Movie_bhaa028

## Notes

The authors are grateful to Dr Nobuhiro Hagura (Center for Information and Neural Networks) and Dr Shu Imaizumi (Ochanomizu University) for their insightful comments. *Conflict of Interest*: None declared.

## References

[ref1] Asai T 2015 Feedback control of one's own action: self-other sensory attribution in motor control. Conscious Cogn. 38:118–129. 26587957 10.1016/j.concog.2015.11.002

[ref2] Asai T 2016 Self is “other”, other is “self”: poor self-other discriminability explains schizotypal twisted agency judgment. Psychiatry Res. 246:593–600. 27836244 10.1016/j.psychres.2016.10.082

[ref3] Asai T 2017 Know thy agency in predictive coding: meta-monitoring over forward modeling. Conscious Cogn. 51:82–99. 28327348 10.1016/j.concog.2017.03.001

[ref4] Astafiev SV, Stanley CM, Shulman GL, Corbetta M 2004 Extrastriate body area in human occipital cortex responds to the performance of motor actions. Nat Neurosci. 7:542–548. 15107859 10.1038/nn1241

[ref5] Bassolino M, Serino A, Ubaldi S, Ladavas E 2010 Everyday use of the computer mouse extends peripersonal space representation. Neuropsychologia. 48:803–811. 19931547 10.1016/j.neuropsychologia.2009.11.009

[ref6] Beyer F, Sidarus N, Fleming S, Haggard P 2018 Losing control in social situations: how the presence of others affects neural processes related to sense of agency. eNeuro. 5:ENEURO.0336-0317.2018. 10.1523/ENEURO.0336-17.2018 PMC5844060 29527568

[ref7] Blakemore SJ, Frith CD, Wolpert DM 2001 The cerebellum is involved in predicting the sensory consequences of action. Neuroreport. 12:1879–1884. 11435916 10.1097/00001756-200107030-00023

[ref8] Blakemore SJ, Rees G, Frith CD 1998 How do we predict the consequences of our actions? A functional imaging study. Neuropsychologia. 36:521–529. 9705062 10.1016/s0028-3932(97)00145-0

[ref9] Blakemore SJ, Wolpert D, Frith C 2000 Why can't you tickle yourself? Neuroreport. 11:R11–R16. 10943682 10.1097/00001756-200008030-00002

[ref10] Blanke O, Slater M, Serino A 2015 Behavioral, neural, and computational principles of bodily self-consciousness. Neuron. 88:145–166. 26447578 10.1016/j.neuron.2015.09.029

[ref11] Bode S, Haynes JD 2009 Decoding sequential stages of task preparation in the human brain. NeuroImage. 45:606–613. 19111624 10.1016/j.neuroimage.2008.11.031

[ref12] Bzdok D, Langner R, Schilbach L, Jakobs O, Roski C, Caspers S, Laird AR, Fox PT, Zilles K, Eickhoff SB 2013 Characterization of the temporo-parietal junction by combining data-driven parcellation, complementary connectivity analyses, and functional decoding. NeuroImage. 81:381–392. 23689016 10.1016/j.neuroimage.2013.05.046 PMC4791053

[ref13] Chambon V, Moore JW, Haggard P 2015 TMS stimulation over the inferior parietal cortex disrupts prospective sense of agency. Brain Struct Funct. 220:3627–3639. 25134684 10.1007/s00429-014-0878-6

[ref14] Chambon V, Sidarus N, Haggard P 2014 From action intentions to action effects: how does the sense of agency come about? Front Hum Neurosci. 8:320. 24860486 10.3389/fnhum.2014.00320 PMC4030148

[ref15] Chambon V, Wenke D, Fleming SM, Prinz W, Haggard P 2013 An online neural substrate for sense of agency. Cereb Cortex. 23:1031–1037. 22510529 10.1093/cercor/bhs059

[ref16] Christoff K, Cosmelli D, Legrand D, Thompson E 2011 Specifying the self for cognitive neuroscience. Trends Cogn Sci. 15:104–112. 21288760 10.1016/j.tics.2011.01.001

[ref17] David N, Cohen MX, Newen A, Bewernick BH, Shah NJ, Fink GR, Vogeley K 2007 The extrastriate cortex distinguishes between the consequences of one's own and others' behavior. NeuroImage. 36:1004–1014. 17478105 10.1016/j.neuroimage.2007.03.030

[ref18] David N, Newen A, Vogeley K 2008 The ``sense of agency'' and its underlying cognitive and neural mechanisms. Conscious Cogn. 17:523–534. 18424080 10.1016/j.concog.2008.03.004

[ref19] Eddy CM 2016 The junction between self and other? Temporo-parietal dysfunction in neuropsychiatry. Neuropsychologia. 89:465–477. 27457686 10.1016/j.neuropsychologia.2016.07.030

[ref20] Ehrsson HH, Holmes NP, Passingham RE 2005 Touching a rubber hand: feeling of body ownership is associated with activity in multisensory brain areas. J Neurosci. 25:10564–10573. 16280594 10.1523/JNEUROSCI.0800-05.2005 PMC1395356

[ref21] Ehrsson HH, Spence C, Passingham RE 2004 That's my hand! Activity in premotor cortex reflects feeling of ownership of a limb. Science. 305:875–877. 15232072 10.1126/science.1097011

[ref22] Farrer C, Franck N, Georgieff N, Frith CD, Decety J, Jeannerod A 2003 Modulating the experience of agency: a positron emission tomography study. NeuroImage. 18:324–333. 12595186 10.1016/s1053-8119(02)00041-1

[ref23] Farrer C, Frey SH, Van Horn JD, Tunik E, Turk D, Inati S, Grafton ST 2008 The angular gyrus computes action awareness representations. Cereb Cortex. 18:254–261. 17490989 10.1093/cercor/bhm050

[ref24] Farrer C, Frith CD 2002 Experiencing oneself vs another person as being the cause of an action: the neural correlates of the experience of agency. NeuroImage. 15:596–603. 11848702 10.1006/nimg.2001.1009

[ref25] Frith CD, Blakemore S, Wolpert DM 2000 Explaining the symptoms of schizophrenia: abnormalities in the awareness of action. Brain Res Rev. 31:357–363. 10719163 10.1016/s0165-0173(99)00052-1

[ref26] Fukushima H, Goto Y, Maeda T, Kato M, Umeda S 2013 Neural substrates for judgment of self-agency in ambiguous situations. PLoS One. 8:e72267. 23977268 10.1371/journal.pone.0072267 PMC3747082

[ref27] Gallagher S 2000 Philosophical conceptions of the self: implications for cognitive science. Trends Cogn Sci. 4:14–21. 10637618 10.1016/s1364-6613(99)01417-5

[ref28] Gentile G, Guterstam A, Brozzoli C, Ehrsson HH 2013 Disintegration of multisensory signals from the real hand reduces default limb self-attribution: an fMRI study. J Neurosci. 33:13350–13366. 23946393 10.1523/JNEUROSCI.1363-13.2013 PMC3742923

[ref29] Grivaz P, Blanke O, Serino A 2017 Common and distinct brain regions processing multisensory bodily signals for peripersonal space and body ownership. NeuroImage. 147:602–618. 28017920 10.1016/j.neuroimage.2016.12.052

[ref30] Guterstam A, Bjornsdotter M, Gentile G, Ehrsson HH 2015 Posterior cingulate cortex integrates the senses of self-location and body ownership. Curr Biol. 25:1416–1425. 25936550 10.1016/j.cub.2015.03.059

[ref31] Guterstam A, Gentile G, Ehrsson HH 2013 The invisible hand illusion: multisensory integration leads to the embodiment of a discrete volume of empty space. J Cogn Neurosci. 25:1078–1099. 23574539 10.1162/jocn_a_00393

[ref32] Haggard P 2017 Sense of agency in the human brain. Nat Rev Neurosci. 18:196. 28251993 10.1038/nrn.2017.14

[ref33] Haggard P, Chambon V 2012 Sense of agency. Curr Biol. 22:R390–R392. 22625851 10.1016/j.cub.2012.02.040

[ref34] Haynes JD 2015 A primer on pattern-based approaches to fMRI: principles, pitfalls, and perspectives. Neuron. 87:257–270. 26182413 10.1016/j.neuron.2015.05.025

[ref35] Haynes JD, Rees G 2005 Predicting the orientation of invisible stimuli from activity in human primary visual cortex. Nat Neurosci. 8:686–691. 15852013 10.1038/nn1445

[ref36] Haynes JD, Sakai K, Rees G, Gilbert S, Frith C, Passingham RE 2007 Reading hidden intentions in the human brain. Curr Biol. 17:323–328. 17291759 10.1016/j.cub.2006.11.072

[ref37] Hebart MN, Baker CI 2018 Deconstructing multivariate decoding for the study of brain function. NeuroImage. 180:4–18. 28782682 10.1016/j.neuroimage.2017.08.005 PMC5797513

[ref38] Ionta S, Heydrich L, Lenggenhager B, Mouthon M, Fornari E, Chapuis D, Gassert R, Blanke O 2011 Multisensory mechanisms in temporo-parietal cortex support self-location and first-person perspective. Neuron. 70:363–374. 21521620 10.1016/j.neuron.2011.03.009

[ref39] Jakobs O, Langner R, Caspers S, Roski C, Cieslik EC, Zilles K, Laird AR, Fox PT, Eickhoff SB 2012 Across-study and within-subject functional connectivity of a right temporo-parietal junction subregion involved in stimulus-context integration. NeuroImage. 60:2389–2398. 22387170 10.1016/j.neuroimage.2012.02.037 PMC3321133

[ref40] James W 1890 The Principles of Psychology. Dover Publications.

[ref41] Kamitani Y, Tong F 2005 Decoding the visual and subjective contents of the human brain. Nat Neurosci. 8:679–685. 15852014 10.1038/nn1444 PMC1808230

[ref42] Koreki A, Maeda T, Okimura T, Terasawa Y, Kikuchi T, Umeda S, Nishikata S, Yagihashi T, Kasahara M, Nagai C et al. 2019 Dysconnectivity of the agency network in schizophrenia: a functional magnetic resonance imaging study. Front Psych. 10:171. 10.3389/fpsyt.2019.00171 PMC6456683 31001152

[ref43] Kriegeskorte N, Goebel R, Bandettini P 2006 Information-based functional brain mapping. Proc Natl Acad Sci USA. 103:3863–3868. 16537458 10.1073/pnas.0600244103 PMC1383651

[ref44] Lakens D 2013 Calculating and reporting effect sizes to facilitate cumulative science: a practical primer for t-tests and ANOVAs. Front Psychol. 4:863. 24324449 10.3389/fpsyg.2013.00863 PMC3840331

[ref45] Langfelder P, Luo R, Oldham MC, Horvath S 2011 Is my network module preserved and reproducible? PLoS Comput Biol. 7:e1001057. 21283776 10.1371/journal.pcbi.1001057 PMC3024255

[ref46] Legrand D 2007 Pre-reflective self-as-subject from experiential and empirical perspectives. Conscious Cogn. 16:583–599. 17533140 10.1016/j.concog.2007.04.002

[ref47] Legrand D, Ruby P 2009 What is self-specific? Theoretical investigation and critical review of neuroimaging results. Psychol Rev. 116:252–282. 19159156 10.1037/a0014172

[ref48] Miall RC, Wolpert DM 1996 Forward models for physiological motor control. Neural Netw. 9:1265–1279. 12662535 10.1016/s0893-6080(96)00035-4

[ref49] Miele DB, Wager TD, Mitchell JP, Metcalfe J 2011 Dissociating neural correlates of action monitoring and metacognition of agency. J Cogn Neurosci. 23:3620–3636. 21563889 10.1162/jocn_a_00052

[ref50] Moore JW, Fletcher PC 2012 Sense of agency in health and disease: a review of cue integration approaches. Conscious Cogn. 21:59–68. 21920777 10.1016/j.concog.2011.08.010 PMC3315009

[ref51] Moore JW, Wegner DM, Haggard P 2009 Modulating the sense of agency with external cues. Conscious Cogn. 18:1056–1064. 19515577 10.1016/j.concog.2009.05.004

[ref52] Mur M, Bandettini PA, Kriegeskorte N 2009 Revealing representational content with pattern-information fMRI—an introductory guide. Soc Cogn Affect Neurosci. 4:101–109. 19151374 10.1093/scan/nsn044 PMC2656880

[ref53] Nahab FB, Kundu P, Gallea C, Kakareka J, Pursley R, Pohida T, Miletta N, Friedman J, Hallett M 2011 The neural processes underlying self-agency. Cereb Cortex. 21:48–55. 20378581 10.1093/cercor/bhq059 PMC3000563

[ref54] Norman KA, Polyn SM, Detre GJ, Haxby JV 2006 Beyond mind-reading: multi-voxel pattern analysis of fMRI data. Trends Cogn Sci. 10:424–430. 16899397 10.1016/j.tics.2006.07.005

[ref55] Ogawa K, Inui T 2007 Lateralization of the posterior parietal cortex for internal monitoring of self-versus externally generated movements. J Cogn Neurosci. 19:1827–1835. 17958485 10.1162/jocn.2007.19.11.1827

[ref56] Pacherie E 2007 The sense of control and the sense of agency. Psyche. 13:1–30.

[ref57] Petkova VI, Bjornsdotter M, Gentile G, Jonsson T, Li TQ, Ehrsson HH 2011 From part- to whole-body ownership in the multisensory brain. Curr Biol. 21:1118–1122. 21683596 10.1016/j.cub.2011.05.022

[ref58] Preston C, Newport R 2008 Misattribution of movement agency following right parietal TMS. Soc Cogn Affect Neurosci. 3:26–32. 19015092 10.1093/scan/nsm036 PMC2569818

[ref59] Ritterband-Rosenbaum A, Karabanov AN, Christensen MS, Nielsen JB 2014 10 Hz rTMS over right parietal cortex alters sense of agency during self-controlled movements. Front Hum Neurosci. 8:471. 25009489 10.3389/fnhum.2014.00471 PMC4070178

[ref60] Schnell K, Heekeren K, Schnitker R, Daumann J, Weber J, Hesselmann V, Moller-Hartmann W, Thron A, Gouzoulis-Mayfrank E 2007 An fMRI approach to particularize the frontoparietal network for visuomotor action monitoring: detection of incongruence between test subjects' actions and resulting perceptions. NeuroImage. 34:332–341. 17046287 10.1016/j.neuroimage.2006.08.027

[ref61] Shibata K, Watanabe T, Kawato M, Sasaki Y 2016 Differential activation patterns in the same brain region led to opposite emotional states. PLoS Biol. 14:e1002546. 27608359 10.1371/journal.pbio.1002546 PMC5015828

[ref62] Soon CS, Brass M, Heinze HJ, Haynes JD 2008 Unconscious determinants of free decisions in the human brain. Nat Neurosci. 11:543–545. 18408715 10.1038/nn.2112

[ref63] Spengler S, von Cramon DY, Brass M 2009 Was it me or was it you? How the sense of agency originates from ideomotor learning revealed by fMRI. NeuroImage. 46:290–298. 19457378 10.1016/j.neuroimage.2009.01.047

[ref64] Sperduti M, Delaveau P, Fossati P, Nadel J 2011 Different brain structures related to self- and external-agency attribution: a brief review and meta-analysis. Brain Struct Funct. 216:151–157. 21212978 10.1007/s00429-010-0298-1

[ref65] Synofzik M, Vosgerau G, Newen A 2008 Beyond the comparator model: a multifactorial two-step account of agency. Conscious Cogn. 17:219–239. 17482480 10.1016/j.concog.2007.03.010

[ref66] Synofzik M, Vosgerau G, Voss M 2013 The experience of agency: an interplay between prediction and postdiction. Front Psychol. 4:127. 23508565 10.3389/fpsyg.2013.00127 PMC3597983

[ref67] Tsakiris M, Longo MR, Haggard P 2010 Having a body versus moving your body: neural signatures of agency and body-ownership. Neuropsychologia. 48:2740–2749. 20510255 10.1016/j.neuropsychologia.2010.05.021

[ref68] Tzourio-Mazoyer N, Landeau B, Papathanassiou D, Crivello F, Etard O, Delcroix N, Mazoyer B, Joliot M 2002 Automated anatomical labeling of activations in SPM using a macroscopic anatomical parcellation of the MNI MRI single-subject brain. NeuroImage. 15:273–289. 11771995 10.1006/nimg.2001.0978

[ref69] Walsh V 2003 A theory of magnitude: common cortical metrics of time, space and quantity. Trends Cogn Sci. 7:483–488. 14585444 10.1016/j.tics.2003.09.002

[ref70] Wenke D, Fleming SM, Haggard P 2010 Subliminal priming of actions influences sense of control over effects of action. Cognition. 115:26–38. 19945697 10.1016/j.cognition.2009.10.016

[ref71] Wisniewski D, Goschke T, Haynes J-D 2016 Similar coding of freely chosen and externally cued intentions in a fronto-parietal network. NeuroImage. 134:450–458. 27107470 10.1016/j.neuroimage.2016.04.044

[ref72] Wisniewski D, Reverberi C, Momennejad I, Kahnt T, Haynes JD 2015 The role of the parietal cortex in the representation of task-reward associations. J Neurosci. 35:12355–12365. 26354905 10.1523/JNEUROSCI.4882-14.2015 PMC6605393

[ref73] Yomogida Y, Sugiura M, Sassa Y, Wakusawa K, Sekiguchi A, Fukushima A, Takeuchi H, Horie K, Sato S, Kawashima R 2010 The neural basis of agency: an fMRI study. NeuroImage. 50:198–207. 20026225 10.1016/j.neuroimage.2009.12.054

