# Supplementary material for: Sense of Agency Beyond Sensorimotor Process: Decoding Self-Other Action Attribution in the Human Brain
Source: Cereb Cortex. 2020 Mar 3;30(7):4076–91. doi: 10.1093/cercor/bhaa028 (PMC7264682; doi:10.1093/cercor/bhaa028)
Supplement: Supplementary_bhaa028 [file supplementary_bhaa028.docx]

Supplementary Information

**Sense of agency beyond sensorimotor process: Decoding self-other action attribution in the human brain**

Ryu Ohata, Tomohisa Asai, Hiroshi Kadota, Hiroaki Shigemasu, Kenji Ogawa, and Hiroshi Imamizu

Correspondence to H.I (imamizu@gmail.com) or R.O (ryu.oohata@gmail.com)

**Supplementary Results**

**Exclusion of participants with low and unstable discriminability of action attribution**

We measured individuals’ self-other discriminability by fitting the linear regression model to each participant’s rating scores (regression lines in Fig. 2). We found some of the participants showing relatively low and unstable discriminability, which could be due to low engagement in the task or schizotypal personality traits as suggested by a previous study (Asai 2016). As an additional analysis, we excluded the participants with poor discriminability from the data and analyzed the remaining data to examine whether the poor discriminability affected our decoding result. First, we systematically determined the exclusion criteria from an independent dataset including 42 participants. We generated an empirical distribution of the slope from the independent dataset. These participants were not enrolled in the current study but carried out the same behavioral task as that in the current study without fMRI scanning (unpublished data). Note that a slope is defined as a coefficient on the morphing ratio in the linear regression model (for details see Results: Self-other rating score on morphing ratio condition). We identified the lower limit of the 99.99% confidence interval (CI) of the slope in the empirical distribution (3.89) as the criterion. In addition, we found the rating score for some participants showed relatively low stability in the most apparent other-attribution (self 0%) condition in comparison with the other morphing ratio conditions. Thus, we also eliminated the participants showing high variability of rating score in self 0% condition. We generated an empirical distribution consisting of the SD of the rating score in the condition using the 42-participant data. Then we added the upper limit of the 99.99% CI of the SD (2.89) to the exclusion criteria. We excluded a total of seven participants according to the above criteria. Supplementary Table S1 denotes the slope and SD values of each participant.

We performed a random-effects group analysis on the smoothed *z*-score maps of the remaining eleven (out of eighteen) participants. Supplementary Fig. S3 shows the clusters in which we could significantly decode self-other attribution from their voxel patterns (red regions in Supplementary Fig. S3, *p* < 0.05 FWE-corrected at cluster level with a cluster-forming threshold of *p* < 0.001; all clusters are reported in Supplementary Table S2). We found the clusters in the bilateral precentral gyrus in the third and fourth cycles and in the right supramarginal gyrus (SMG) in the fifth cycle of the Move period. This result was similar to that including the seven participants (eighteen participants in total) except a few points as follows. The number of clusters decreased from 15 to 12. We found no clusters in the second cycle but newly found clusters in the left middle temporal gyrus and right inferior occipital gyrus in the fourth cycle. Therefore, with the exclusion of the above participants, we were able to confirm our main finding that the sensorimotor, higher visual cortices and posterior parietal regions contain information that could predict the self-other attribution.

**Mass univariate analysis of voxel-wise activation modulated by self-other attribution**

We conducted mass univariate analysis based on the general linear model to examine whether the activation level of brain regions could be explained by the difference in self-other attribution. For each run, the trial-by-trial rating score and the five morphing conditions were entered into the model as two parametric regressors of the 10-s Move period, each of which was modeled as a 10-s boxcar function and convolved with the canonical hemodynamic response function as implemented in SPM8. Note that five morphing conditions were converted into values ranging from 1 (corresponding to self 0% condition) to 5 (corresponding to self 100% condition). We additionally included four boxcar functions, each of which modeled the remaining conditions (i.e., the Cue, Ready, Delay and Rate periods). Single-subject contrast images were generated using the first-level fixed-effects analysis. Then, we took them into the second-level analysis using a random-effects model of a one-sample *t*-test. Note that unlike the multi-voxel pattern analysis (MVPA), spatial smoothing was applied to the data with a 6-mm full-width at half-maximum (FWHM) Gaussian kernel for this mass univariate analysis.

**Modulation by rating score**: As a result, we found the bilateral putamen, right postcentral gyrus, supplementary motor area (SMA), and cerebellar vermis lobule VII as the regions whose activity was positively modulated with participants’ trial-by-trial rating score (red areas in Supplementary Fig. S6A, *p* < 0.05 family-wise error (FWE) corrected at cluster level with a cluster-forming threshold of *p* < 0.001; all clusters are also reported in Supplementary Table S3). Positive modulation indicates that the more likely the participants judged that the cursor movement was attributed to their own cursor movement, the greater the activation in the cluster became. By contrast, we found no significant cluster in which activity was negatively modulated with rating score.

**Modulation by morphing condition**: Next, we explored the region whose activity was modulated with the five morphing conditions. Activity in the anterior part of the cingulate gyrus was positively modulated with the morphing conditions (red area in Supplementary Fig. S6B, *p* < 0.05 FWE corrected at cluster level with a cluster-forming threshold of *p* < 0.001), while the left SMG, right middle frontal gyrus, right superior temporal gyrus, SMA and cerebellar cortical crus II were found as the regions in which the activity was negatively modulated with the morphing conditions (blue areas in Supplementary Fig. S6B, *p* < 0.05 FWE corrected at cluster level with a cluster-forming threshold of *p* < 0.001**)**. Positive modulation indicates that the more self-movement was included in the cursor movement (i.e., the less other-movement was included in the cursor movement), the greater the activation in the cluster became. On the other hand, negative modulation means that the more self-movement was included, the less activation was found.

There was a possibility that the MVPA identified the clusters shown in Fig. 5 because the rating score modulated a uniform activation level, rather than the multivoxel patterns, in the clusters. However, the clusters found by the univariate analysis (Supplementary Fig. S6A) did not overlap those identified by the MVPA (Fig. 5). This univariate result negated the possibility that our MVPA result could be explained only by the activation level in the found regions.

**Supplementary Figures**


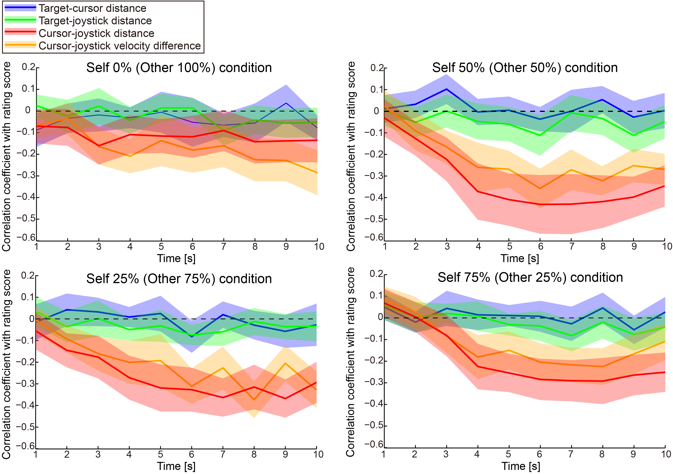


**Supplementary Figure S1.** Relationship between tracing behavior and self-other attribution scores (see also Figure 4B). Time courses of Fisher-transformed correlation coefficients between each behavioral measure and self-other attribution scores in data of all conditions (except for self 100% condition). Colored lines indicate the distance (target-cursor: blue, target-joystick: green, cursor-joystick: red) and velocity difference (cursor-joystick: orange). See Figure 4A for definition of each behavioral measure. Shaded areas denote 95% confidence intervals.


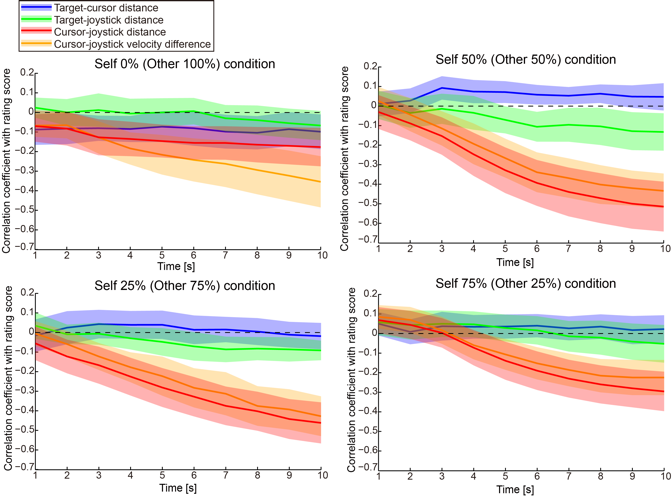


**Supplementary Figure S2.** Relationship between accumulated value of tracing behavior and self-other attribution scores (see also Figure 4C). Time courses of Fisher-transformed correlation coefficients between accumulated values of each behavioral measure and self-other attribution scores in data of all conditions (except for self 100% condition). Colored lines indicate the distances (target-cursor: blue, target-joystick: green, cursor-joystick: red) and the velocity difference (cursor-joystick: orange). See Figure 4A for definition of each behavioral measure. Shaded areas denote 95% confidence intervals.


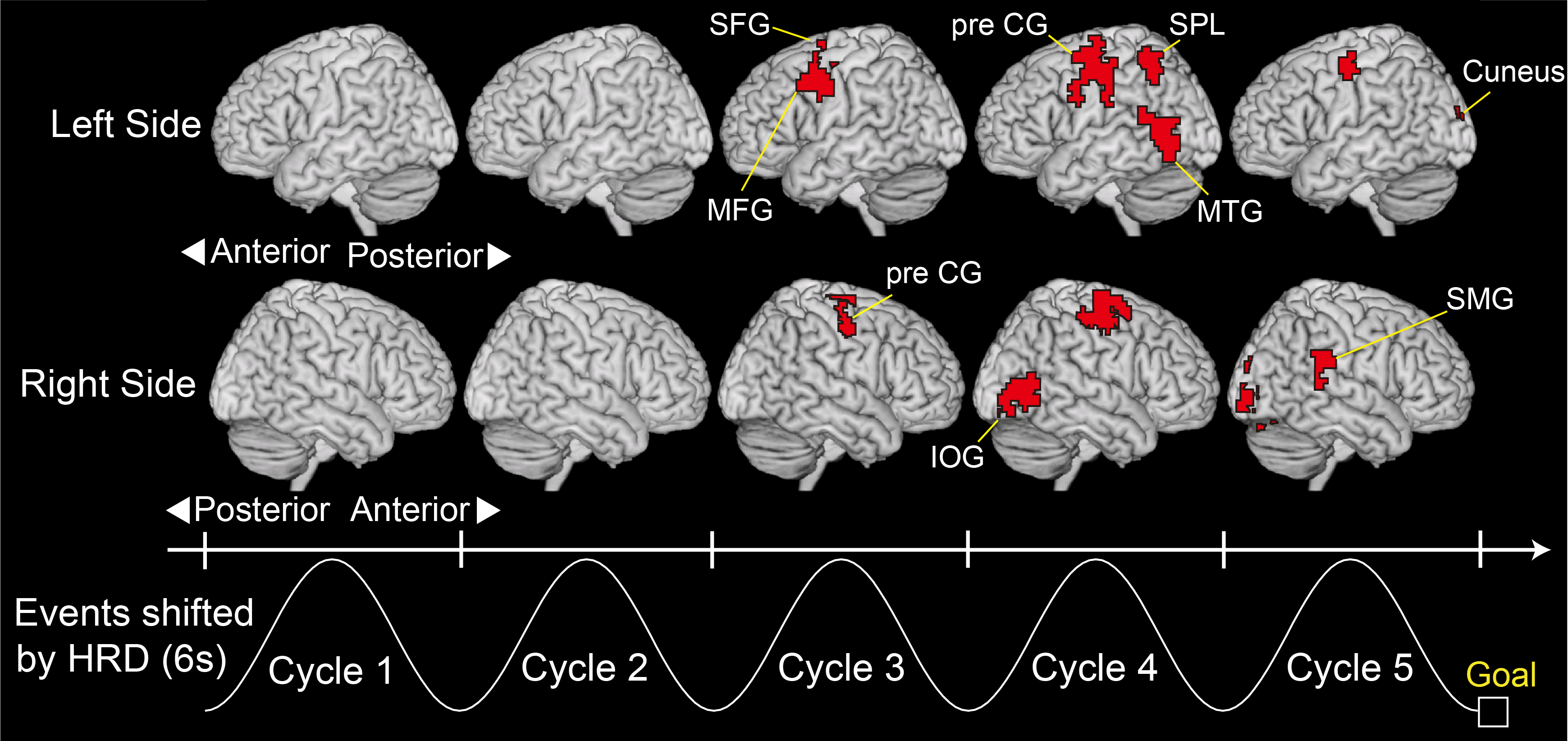


**Supplementary Figure S3.** Decoding performance for self-other attribution during movement while taking into account the effect of low and unstable discriminability of self-other attribution. A searchlight decoding analysis was applied to a volume scanned every 2 s during the 10-s Move period to create accuracy maps. A random-effects group analysis was performed on the smoothed accuracy maps of the eleven (out of eighteen) participants exceeding the criteria. The red areas denote the clusters of significant decoding accuracy (*p* < 0.05 FWE-corrected at cluster level with a cluster-forming threshold of *p* < 0.001). The sinusoidal waves represent a typical cursor movement along the timeline shifted by 6 s from the actual time considering the hemodynamic response delay (HRD). IOG: inferior occipital gyrus, MFG: middle frontal gyrus, MTG: middle temporal gyrus, pre CG: precentral gyrus, SFG: superior frontal gyrus, SMG: supramarginal gyrus, SPL: superior parietal lobe.


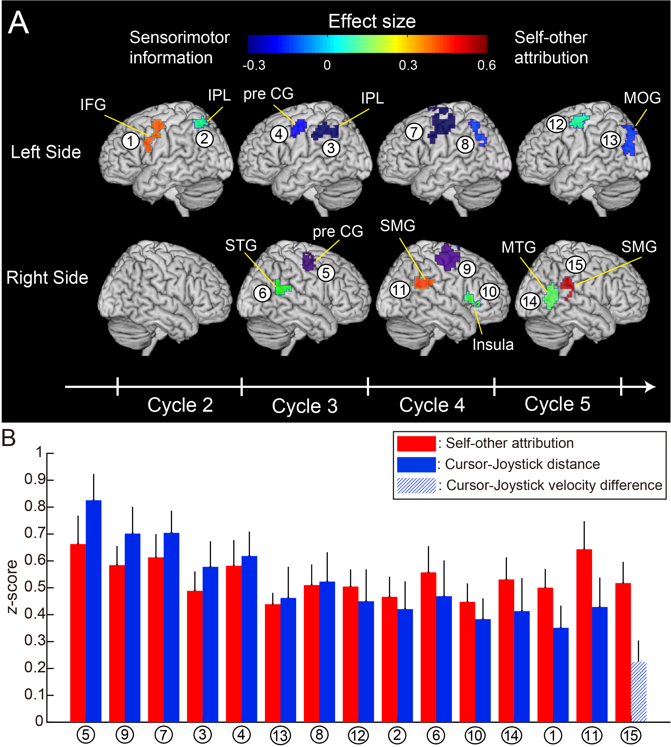


**Supplementary Figure S4.** (A) Clusters showing significant decoding performance for self-other attribution (Fig. 5). Colors represent the effect sizes for the difference between decoding performance for self-other attribution and that for sensorimotor information. This figure is identical to Fig. 7A. (B) Decoding performances (*z*-scores) for self-other attribution (red bars) and sensorimotor information (blue bars) in the 15 clusters. We compared the *z*-score for cursor-joystick distance with that for velocity difference in each cluster and chose the higher score as the decoding performance for sensorimotor information. Blue solid and shaded bars denote decoding performance for cursor-joystick distance and velocity difference, respectively. Numbers correspond to those in (A). Clusters are sorted according to the effect size in ascending order from negative to positive values (same order as Fig. 7B). Error bars show standard error of the mean.


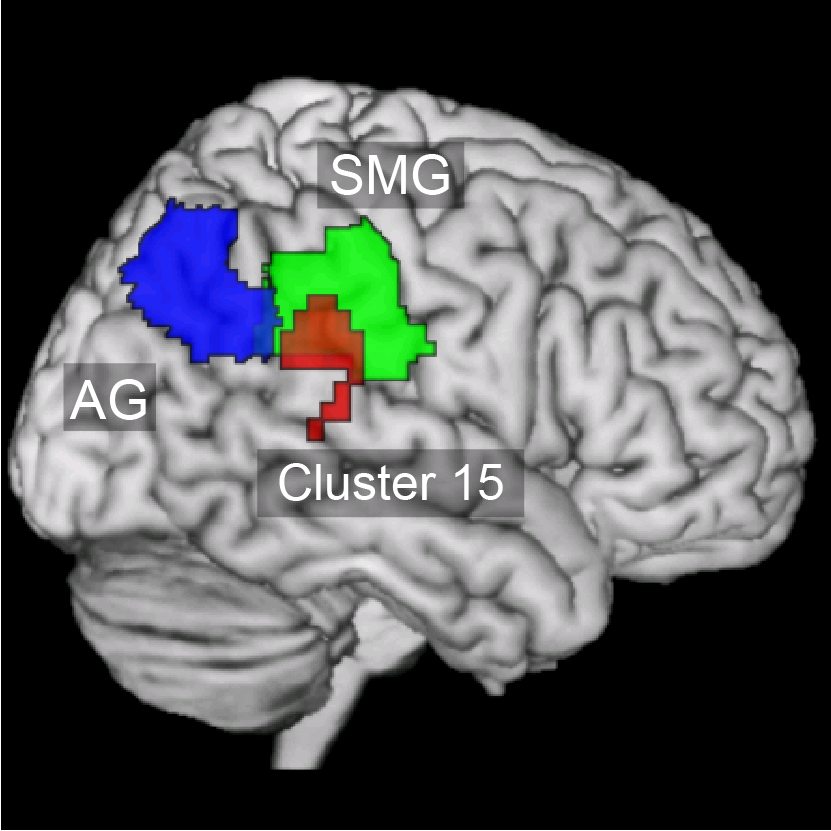


**Supplementary Figure S5.** Anatomical location of the SMG (supramarginal gyrus) cluster found by multi-voxel pattern regression. The red area indicates the SMG cluster in the fifth cycle (cluster 15 in Fig. 7A). The blue area indicates the right angular gyrus (AG), and the green area indicates the SMG according to the automated anatomical labeling atlas.


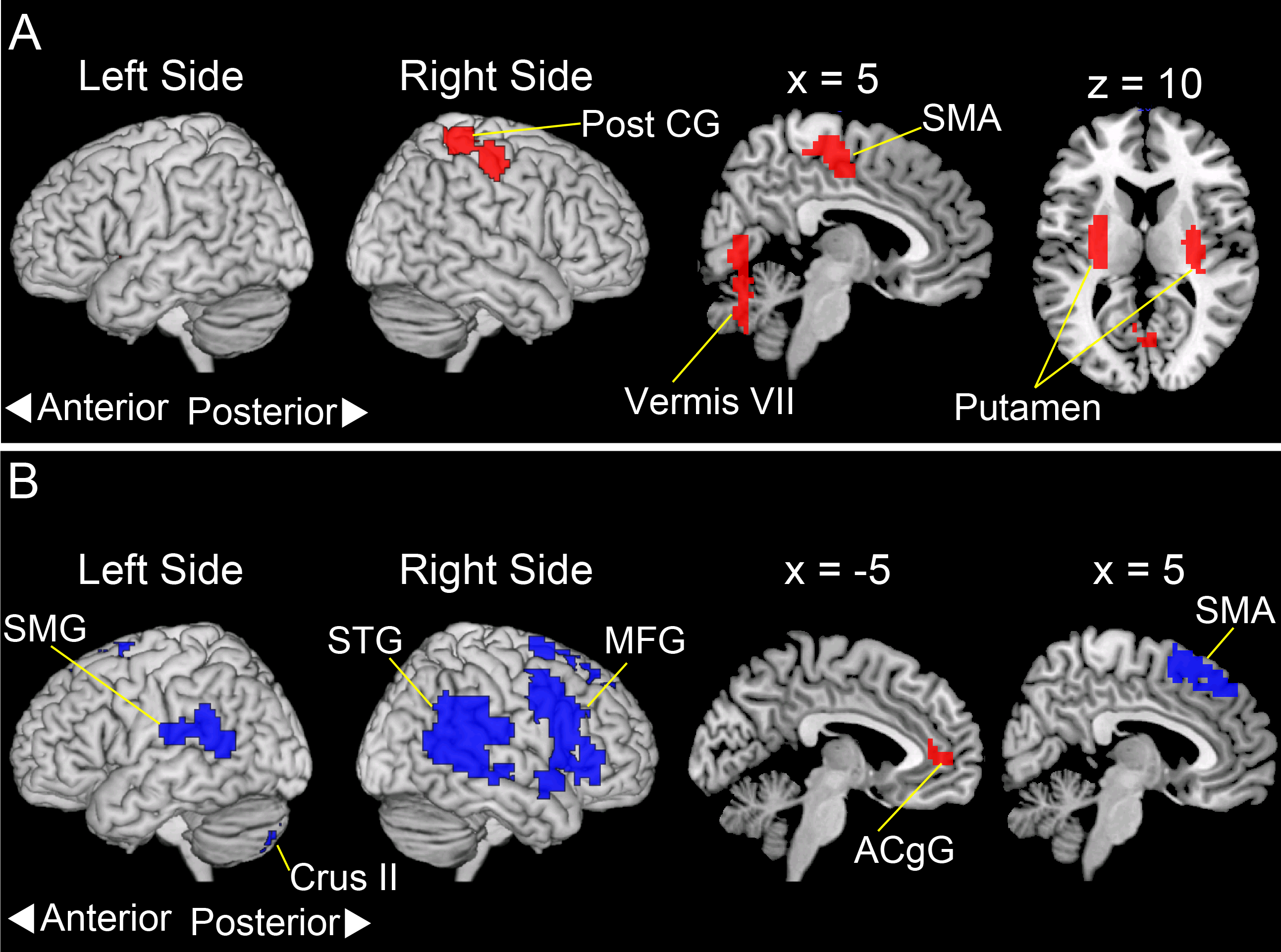


**Supplementary Figure S6.** Mass-univariate analysis result during the Movement period. (A) Clusters (in red) of activation that were positively modulated by self-other rating score. Positive modulation indicates that the more likely the participants judged that the cursor movement was attributed to their own movement, the greater the activation of the cluster became. (B) Clusters of activation that were positively (colored in red) and negatively (colored in blue) modulated by morphing conditions. Positive modulation indicates that the more self-movement was included in the cursor movement, the greater the activation of the cluster became. In contrast, negative modulation indicates that the less self-movement was included in the cursor movement, the greater the activation of the cluster became. A threshold at *p* < 0.05 (FWE-corrected at cluster level with a cluster-forming threshold of *p* < 0.001) was set for statistical testing. ACgG: Cingulate gyrus, anterior part, MFG: middle frontal gyrus, Post CG: postcentral gyrus, SMA: supplementary motor area, SMG: supramarginal gyrus, STG: superior temporal gyrus.

**Supplementary Tables**

**Supplementary Table S1. Slope value of regression line and standard deviation (SD) of rating scores in self 0% condition.**

| Participant | Slope | SD |
| --- | --- | --- |
| 1 | 6.33 | 1.58 |
| 2 | 5.41 | 1.91 |
| 3 | 4.05 | 2.05 |
| 4 | 4.99 | 1.79 |
| 5 | 4.23 | 2.62 |
| 6 | 8.37 | 1.11 |
| 7 | 6.26 | 1.45 |
| 8 | 6.37 | 1.16 |
| 9 | 5.6 | 1.35 |
| 10 | 4.65 | 1.46 |
| 11 | 5.65 | 1.59 |
| 12 | 4.41 | 2.92‡ |
| 13 | 3.49† | 2.13 |
| 14 | 3.15† | 1.70 |
| 15 | 1.74† | 2.55 |
| 16 | 5.11 | 2.90‡ |
| 17 | 3.27† | 3.02‡ |
| 18 | 4.29 | 3.68‡ |

The participants under the horizontal dotted line (i.e., Participants 12~18) were eliminated from the analysis due to low slope values (< 3.89, marked with †) and/or large SDs (> 2.89, marked with ‡).

**Supplementary Table S2. Summary of searchlight decoding of self-other attribution of movement during Move period for eleven participants**

|  |  |  | MNI coordinates  (peak voxel) | | |
| --- | --- | --- | --- | --- | --- |
| Brain region | Side | Cluster size | x | y | z |
| Cycle 3 | | |  |  |  |
| 1. Middle frontal gyrus | Left | 170 | -45 | 8 | 46 |
| 2. Superior frontal gyrus | Left | 68 | -12 | -7 | 62 |
| 3. Precentral gyrus | Right | 159 | 15 | -22 | 70 |
| Cycle 4 | |  |  |  |  |
| 4. Precentral gyrus | Left | 248 | -30 | -4 | 58 |
| 5. Middle temporal gyrus | Left | 201 | -51 | -64 | -2 |
| 6. Superior parietal lobe | Left | 84 | -39 | -49 | 58 |
| 7. Inferior occipital gyrus | Right | 172 | 30 | -76 | 2 |
| 8. Precentral gyrus | Right | 310 | 30 | -10 | 54 |
| Cycle 5 | |  |  |  |  |
| 9. Precentral gyrus | Left | 64 | -36 | -10 | 50 |
| 10. Cuneus | Left | 122 | -9 | -88 | 22 |
| 11. Supramarginal gyrus | Right | 56 | 57 | -37 | 26 |
| 12. Inferior occipital gyrus | Right | 164 | 27 | -91 | 2 |

A threshold at *p* < 0.05 (FWE-corrected at cluster level with a cluster-forming threshold of *p* < 0.001) was set for statistical testing. Cycles correspond to those illustrated at the bottom of Figure 4. They are shifted by 6 s from the actual time considering the HRD.

**Supplementary Table S3. Summary of mass-univariate analysis results**

|  |  |  | MNI coordinates  (peak voxel) | | |
| --- | --- | --- | --- | --- | --- |
| Brain region | Side | Cluster size | x | y | z |
| Positively correlated with rating score | | |  |  |  |
| 1. Putamen | Left | 267 | -24 | -1 | -6 |
| 2. Vermis VII | Bilateral | 290 | 0 | -70 | -30 |
| 3. Supplementary motor area | Bilateral | 205 | 6 | -19 | 58 |
| 4. Postcentral gyrus | Right | 190 | 45 | -19 | 50 |
| 5. Putamen | Right | 118 | 30 | -22 | 10 |
| Negatively correlated with rating score: ns  Positively correlated with morphing condition | |  |  |  |  |
| 1. Cingulate gyrus, anterior part | Left | 67 | -12 | 50 | 2 |
| Negatively correlated with morphing condition | |  |  |  |  |
| 1. Supramarginal gyrus | Left | 211 | -63 | -46 | 26 |
| 2. Cerebellum crus II | Left | 55 | -12 | -79 | -42 |
| 3. Supplementary motor area | Bilateral | 416 | 9 | 11 | 58 |
| 4. Superior temporal gyrus | Right | 744 | 57 | -43 | 18 |
| 5. Middle frontal gyrus | Right | 632 | 42 | 11 | 46 |

A threshold at *p* < 0.05 (FWE-corrected at cluster level with a cluster-forming threshold of p < 0.001) was set for statistical testing. We could not find a significant cluster for cycle 1, 2 or 5 at the same threshold.

**Supplementary Movie**

**Supplementary Movie S1.** Examples of cursor movements displayed to participants. The movie shows five trials in order of self-movement ratio: 75%, 100%, 25%, 0%, and 50%. The movie shows only the 5-s Ready and 10-s Move periods (Fig. 2A). The trial number and self-movement ratio were inserted at the beginning of each trial (on a white background) for display purposes, but these were not shown to participants. The display’s actual refresh ratio was 60 Hz, but in this movie, it was down-sampled to 30 Hz.

**Reference**

Asai T. 2016. Self is “other”, other is “self”: poor self-other discriminability explains schizotypal twisted agency judgment. Psychiatry Res. 246:593-600.
